# Identification of bovine respiratory disease through the nasal microbiome

**DOI:** 10.1186/s42523-022-00167-y

**Published:** 2022-02-22

**Authors:** Ruth Eunice Centeno-Martinez, Natalie Glidden, Suraj Mohan, Josiah Levi Davidson, Esteban Fernández-Juricic, Jacquelyn P. Boerman, Jon Schoonmaker, Deepti Pillai, Jennifer Koziol, Aaron Ault, Mohit S. Verma, Timothy A. Johnson

**Affiliations:** 1 Department of Animal Science, Purdue University, West Lafayette, IN USA; 2 Department of Agricultural and Biological Engineering, Purdue University, West Lafayette, IN USA; 3 Department of Biological Sciences, Purdue University, West Lafayette, IN USA; 4 Department of Comparative Pathobiology, Purdue University, West Lafayette, IN USA; 5 Department of Veterinary Clinical Science, Purdue University, West Lafayette, IN USA; 6 Department of Electrical and Computer Engineering, Purdue University, West Lafayette, IN USA; 7 Weldon School of Biomedical Engineering, Purdue University, West Lafayette, IN USA; 8 Birck Nanotechnology Center, Purdue University, West Lafayette, IN USA; 9 School of Veterinary Medicine, Texas Tech University, Amarillo, TX USA

**Keywords:** Bovine respiratory disease, 16S rRNA gene, qPCR, Cattle nasal microbiome

## Abstract

**Background:**

Bovine respiratory disease (BRD) is an ongoing health and economic challenge in the dairy and beef cattle industries. Multiple risk factors make an animal susceptible to BRD. The presence of *Mannheimia haemolytica*,* Pasteurella multocida*,* Histophilus somni*, and *Mycoplasma bovis* in lung tissues have been associated with BRD mortalities, but they are also commonly present in the upper respiratory tract of healthy animals. This study aims to compare the cattle nasal microbiome (diversity, composition and community interaction) and the abundance of BRD pathogens (by qPCR) in the nasal microbiome of Holstein steers that are apparently healthy (Healthy group, n = 75) or with BRD clinical signs (BRD group, n = 58). We then used random forest models based on nasal microbial community and qPCR results to classify healthy and BRD-affected animals and determined the agreement with the visual clinical signs. Additionally, co-occurring species pairs were identified in visually BRD or healthy animal groups.

**Results:**

Cattle in the BRD group had lower alpha diversity than pen-mates in the healthy group. Amplicon sequence variants (ASVs) from *Trueperella pyogenes*,* Bibersteinia* and *Mycoplasma* spp. were increased in relative abundance in the BRD group, while ASVs from *Mycoplasma bovirhinis* and *Clostridium *sensu stricto were increased in the healthy group. Prevalence of *H. somni* (98%) and *P. multocida* (97%) was high regardless of BRD clinical signs whereas *M. haemolytica* (81 and 61%, respectively) and *M. bovis* (74 and 51%, respectively) were more prevalent in the BRD group than the healthy group. In the BRD group, the abundance of *M. haemolytica* and *M. bovis* was increased, while *H. somni* abundance was decreased. Visual observation of clinical signs agreed with classification by the nasal microbial community (misclassification rate of 32%) and qPCR results (misclassification rate 34%). Co-occurrence analysis demonstrated that the nasal microbiome of BRD-affected cattle presented fewer bacterial associations than healthy cattle.

**Conclusions:**

This study offers insight into the prevalence and abundance of BRD pathogens and the differences in the nasal microbiome between healthy and BRD animals. This suggests that nasal bacterial communities provide a potential platform for future studies and potential pen-side diagnostic testing.

**Supplementary Information:**

The online version contains supplementary material available at 10.1186/s42523-022-00167-y.

## Background

Bovine respiratory disease (BRD) affects the health of beef and dairy cattle of all ages by compromising the immune system and causing morbidity and mortality [1, 2]. The economic impact of BRD treatment in the beef industry has been estimated to be $800–$900 million annually in the US related to animal death, reduction of feed efficiency, and treatment costs [3]. BRD is responsible for approximately 75% of the morbidity and 57% of mortality in US feedlots [4]. In addition, studies have identified that multiple predisposing (e.g. animal age, handling stress and transport) and environmental (stocking density, ambient temperature, humidity and ventilation) risk factors can make an animal susceptible to the action of epidemiological factors (e.g. bacteria, viruses, fungi) and the onset of BRD [5–7]. Within the epidemiological factors, the bacterial species *Mannheimia haemolytica*,* Pasteurella multocida*,* Histophilus somni*, and *Mycoplasma bovis* have been associated with BRD mortalities [8, 9]. Since multiple pathogens are able to cause disease, both the diagnosis and treatment of sick animals are more difficult without additional specific information regarding the cause of illness in each animal.

The bovine respiratory tract possesses multiple lines of defense against pathogen colonization (e.g. mucosal layer of the respiratory tract, mucosal epithelium, and network signaling and communication). These defenses regulate microbial homeostasis between commensals and pathogenic bacteria, clear potential pathogens, and recruit immune cells to protect animal health [10–12]. Nevertheless, when the animals suffer from stress, these defenses can fail, provoking the movement of bacterial pathogens from the lumen into the lung cells, initiating BRD [13, 14]. Most producers rely on animal behavior and appearance observations to identify cattle to be treated for respiratory disease. However, the observation of clinical signs has been shown to have a low sensitivity (62%) and low specificity (63%) to differentiate animals with or without BRD [15–17]. Additional methods have been studied to help identify animals with BRD, such as quantification of acute-phase proteins, white blood cell count, neutrophil/lymphocyte ratios, thoracic ultrasonography, and metabolomics [18–23]. However, further evaluation is needed to confirm the sensitivity, specificity, and accuracy of each method.

In recent years, the utilization of 16S rRNA gene sequencing and quantitative PCR (qPCR) to characterize the cattle respiratory microbiome has increased as a way to understand the respiratory tract microbial ecology associated with BRD. The advantage of 16S rRNA sequencing is that it characterizes the microbial community and provides the relative abundance of individual taxa, while also allowing for the parallel sequencing of multiple samples at the same time [24, 25]. The implementation of qPCR allows the quantification of BRD pathobionts in the cattle respiratory tract [26–28]. For example, Thomas et al. [28], quantified the presence of *P. multocida*,* H. somni* and *M. haemolytica* from nasal swabs collected from healthy cattle using qPCR assays. These bacteria are considered pathobionts because they exhibit both commensal and opportunistic pathogen behavior. It seems if sufficient pressure is exerted on the animal by predisposing factors that the abundance of the pathobionts increases in the upper respiratory tract and the pathobionts are then able to migrate and colonize the lung [29, 30]. Others have quantified the abundance of BRD pathbionts in BRD-affected animals compared to clinically healthy animals. Nonetheless, the difference in the abundance of the pathobionts was attributed to the stress suffered by the animals after being transported and not indicative of disease [31, 32]. In addition, there is a hypothesis that the resident microbial community in the respiratory tract might enhance or prevent BRD pathobionts from increasing in abundance, influencing the likelihood of infection [33, 34]. The genera *Lactobacillus*,* Streptococcus*, and *Enterococcus* were shown to inhibit the growth of *M. haemolytica* when using culture-based methods [35–37].

Even though intensive research has been undertaken to characterize the animal’s respiratory tract, characterization of the lower respiratory tract (LRT) is challenging because it depends on lung tissue collection rather than extracting DNA from swabs [26, 38]. On the contrary, the upper respiratory tract (URT) can be easily sampled with nasal or nasopharyngeal swabs [39]. Correlations in specific taxa between the URT and LRT suggest that there is a mutualistic interrelationship between the two microbial communities [40] and characterizing the nasal microbiome could be used to predict the microbiome in the LRT. Thus, the current observational study aims to characterize the nasal microbiome of cattle with BRD clinical signs and their apparently healthy pen-mates by collecting nasal swabs and quantify the bacterial community diversity as well as to quantify the presence of the four BRD pathobionts. With the results obtained from the study, we expected to demonstrate that the abundance of the BRD pathobionts would be enriched in the nasal cavity of cattle presenting with BRD clinical signs. In the future, characterization of the nasal microbiome could be used to provide additional information for therapeutic decision-making in cattle.

## Results

### Nasal microbiome alpha diversity

DNA extracted from nasal swabs of 124 samples collected from cattle pen-mates were sequenced targeting the V4 region of the 16S rRNA gene. A total of 15,287,698 sequences were identified before the denoising step (DADA2) and 13,127,373 sequences after denoising. A total of 18,010 amplicon sequence variants (ASVs) were observed in the study. After rarefying the total number of reads to 40,420 per sample, 16,376 different ASVs remained and were used to quantify the nasal alpha and beta diversity.

**Fig. 1:**
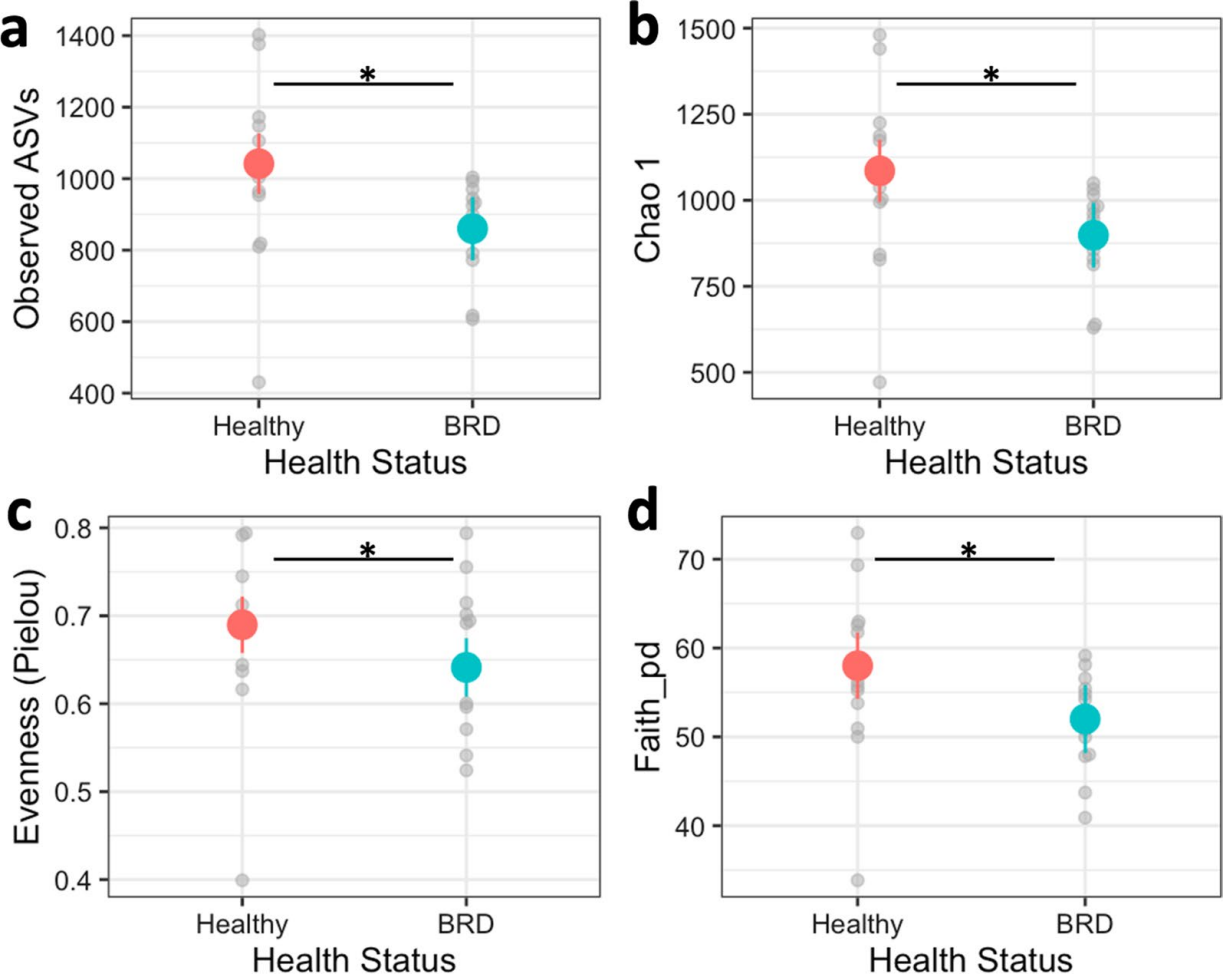
Alpha diversity of the nasal microbiome in cattle that are apparently healthy or display BRD clinical signs (BRD). Observed ASVs (**a**) and Chao 1 (**b**) measure the richness of the microbiome community. Evenness was measured with Pielou (**c**), and the phylogenetic relationship was measured with Faith’s PD (**d**). An asterisk (*) and horizontal line represent a statistical difference (*p* ≤ 0.05) between the two groups. Colored circles and lines represent the means and standard error of the BRD and healthy groups, respectively, and the gray dots represent the raw data of each group

In this study, the richness estimated by Observed ASVs (F_1, 114.18_ = 13.375, *p* < 0.0001; Fig. 1a) and Chao 1 (F_1, 114.38_ = 12.0456, *p* < 0.0001, Fig. 1b), evenness, estimated by Pielou, (F_1, 113.67_ = 7.3700, *p* < 0.007; Fig. 1c), and phylogenetic diversity, estimated by Faith’s PD (F_1, 112.82_ = 10.212, *p* < 0.001; Fig. 1d), were all significantly lower in BRD animals compared to healthy animals.

### Bovine nasal microbiome beta diversity

**Fig. 2:**
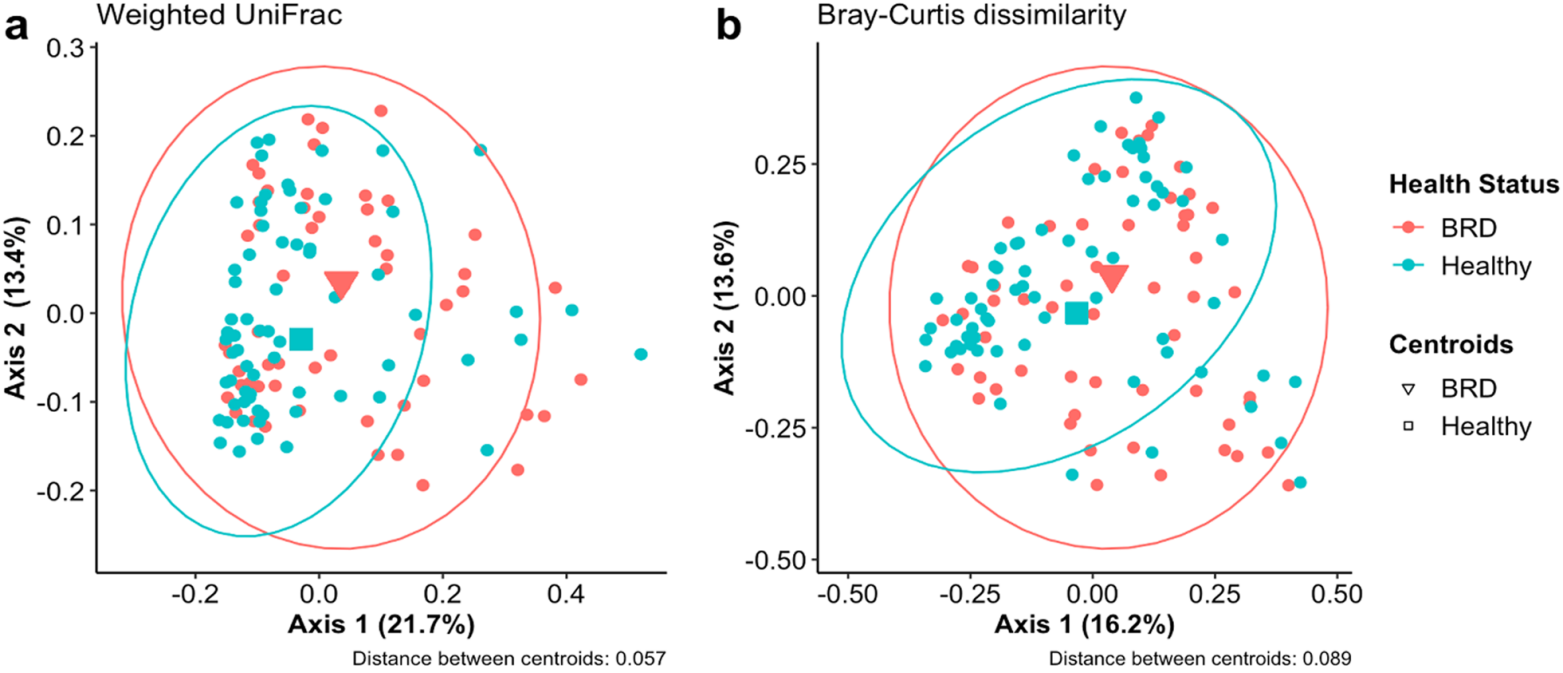
Principal coordinate analysis (PCoA) of Weighted UniFrac distances (**a**) and Bray–Curtis dissimilarity (**b**) between BRD and healthy animals. Ellipses indicate a 95% confidence interval of individuals belonging to each health status group. Axis 1 represents the axis that explains the greatest amount of the variation followed by Axis 2. Larger points indicate the centroids of the ellipses. Distances of the centroids between the two groups are indicated in the caption below each plot

The nasal community structure or distance between the BRD and healthy group (beta diversity) as determined by Weighted UniFrac (F_1, 123 _= 1.83, R^2^ = 0.148,* p* < 0.03; Fig. 2a) and Bray–Curtis dissimilarity (F_1, 123_ = 2.1804, R^2^ = 0.175, *p* < 0.007; Fig. 2b), was significantly different between the two groups. In addition, the dispersion of the samples from the treatment group centroid was not significantly different (*p* > 0.05, see Additional File 1: Table S1). The distance between the group centroids was fairly small, 0.089 (Bray–Curtis) or 0.057 (Weighted UniFrac), indicating limited differences in their microbiome community structure.

### Cattle nasal microbiota taxonomical composition based on 16S rRNA amplicon sequencing

In this study, the top four most relative abundant phyla in the nasal microbiome from all animals were *Proteobacteria* (~ 30% of the community on average), *Firmicutes* (~ 20%), *Bacteroidetes* (~ 20%), and *Actinobacteria* (~ 10%) regardless of the health status. At the family level, *Moraxellaceae* (22%), *Pasteurellaceae* (19%), and *Corynebacteriacea* (10%) had the highest relative abundance regardless of health status; followed by *Mycoplasmataceae* (3.5%) in BRD animals and *Weeksellaceae* (3.7%) in healthy animals. Interestingly, the four genera with the highest relative abundance in BRD animals were *Mannheimia* (5.2%), *Moraxella* (4.6%), *Mycoplasma* (3.9%) and *Corynebacterium* 1 (2.8%), while the most relatively abundant genera in healthy animals were *Corynebacterium* 1 (4.8%), *Moraxella* (4.5%), *Mannheimia* (4.1%) and *Pasteurella* (4%) (see Additional File 1: Fig. S4).

**Fig. 3:**
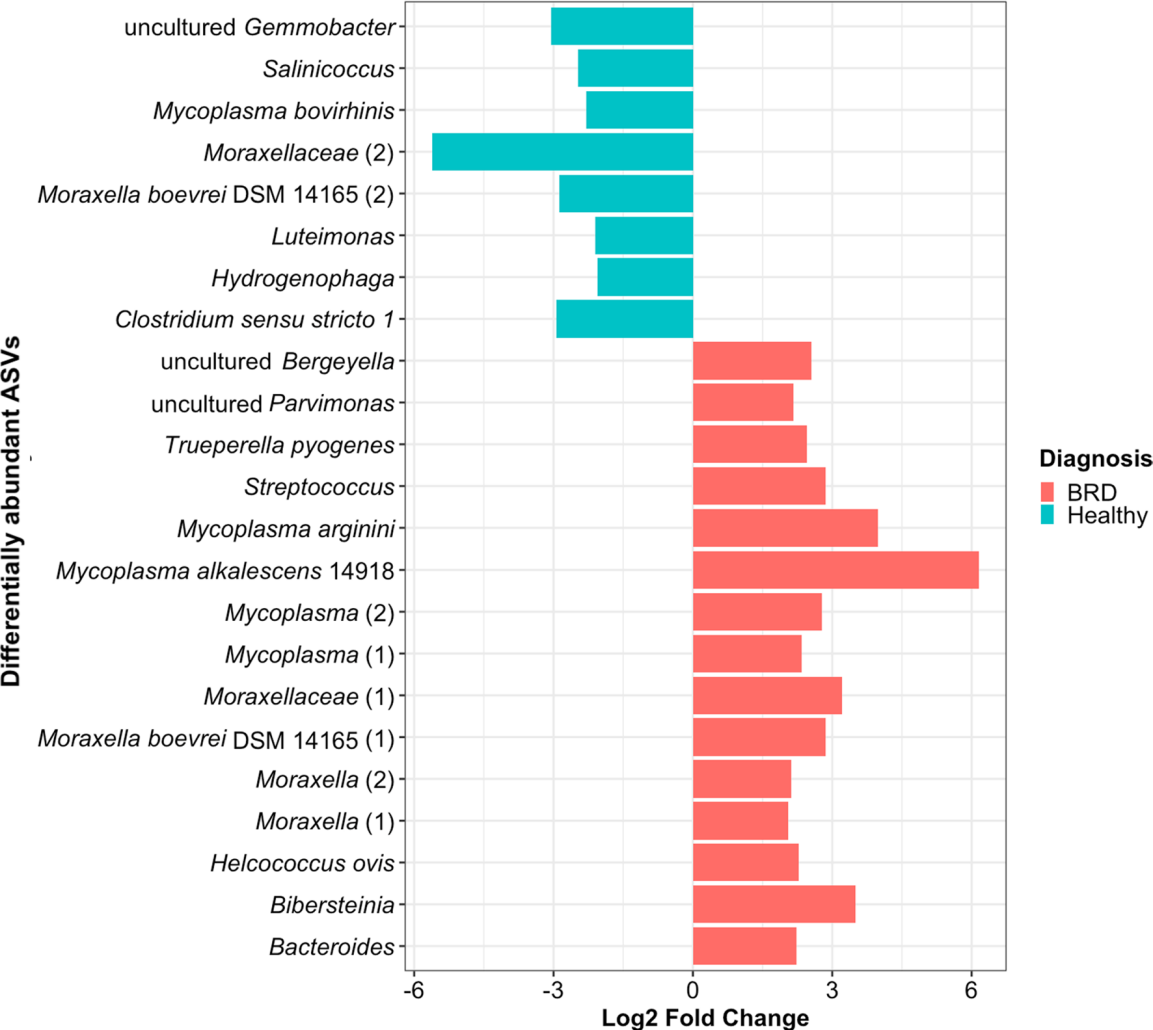
Differentially abundant taxa (ASVs) between animals with BRD clinical signs (BRD) and healthy animals. Bar plot shows the taxa with a log_2_ fold change greater than 2 or less than − 2 and *p* ≤ 0.05. Those with a log_2_ fold change > 2 were those enriched in BRD animals, while a log_2_ fold change < − 2 were those decreased in the BRD animals. Taxa names contain numbers in parenthesis if multiple ASVs were assigned the same taxonomy

DESeq analysis was used to identify differentially abundant taxa in this study. A total of 15 ASVs were increased (log_2_ fold change > 2, *p* ≤ 0.05), and 8 ASVs were decreased in relative abundance in BRD compared to healthy animals (Fig. 3). From the differentially abundant ASVs, the species *Mycoplasma alkalences* 14,918 and *Mycoplasma arginini* had the largest log_2_ fold increase, 6.16 and 3.97, in the BRD group compared to the healthy group. On the contrary, ASVs classified as unclassified *Moraxellaceae*, and *Gemmobacter* had the largest log_2_ fold decrease (5.61 and 3.05) in the BRD group compared to the healthy group. There were 4 ASVs identified as members of the *Mycoplasma* genera that were increased in the BRD group compared to the healthy group, while one *Mycoplasma* ASV (*Mycoplasma bovirhinis*) was decreased in the BRD group.

### Prevalence and quantification of BRD pathobionts by qPCR

**Fig. 4:**
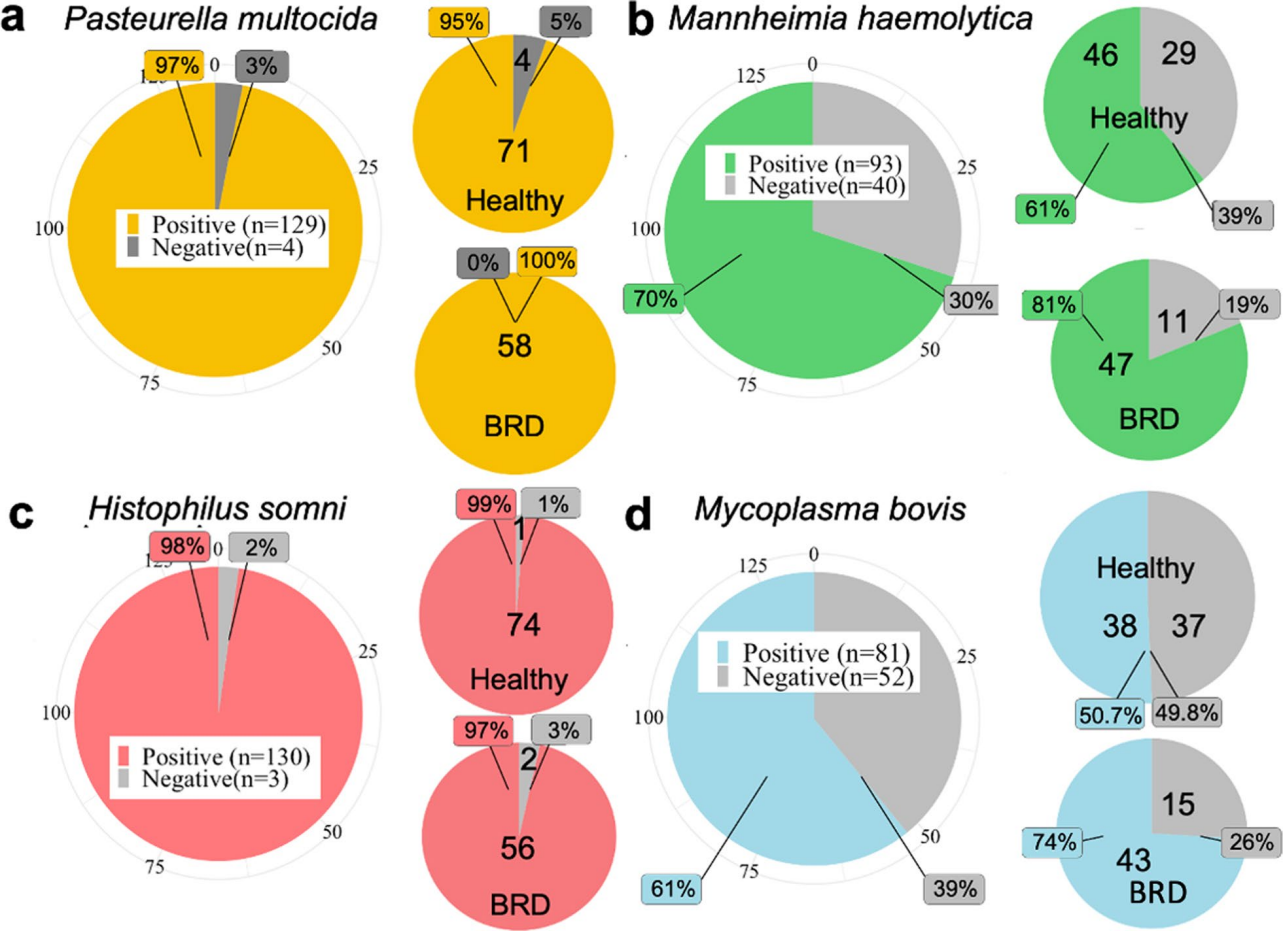
Prevalence of BRD pathobionts in the nasal microbiota of Holstein steers (n = 133) and between healthy (n = 75) and BRD (n = 58) Holstein steer pen-mates according to clinical signs. Prevalence of *Pasteurella multocida* (**a**), *Mannheimina haemolytica* (**b**)*, Histophilus somni* (**c**) and *Mycoplasma bovis* (**d**)

Quantification of *P. multocida*,* H. somni*,* M. haemolytica*,* M. bovis* and total 16S rRNA was performed by qPCR using the DNA extracted from nasal swabs collected from BRD animals and healthy pen-mates. *H. somni* (98%, 129 samples out of 133) and *P. multocida* (97%, 130 out of 133 samples) had higher prevalence regardless of clinical signs of BRD in all the samples, relative to *M. bovis* (61%, 93 out of 133 samples) and *M. haemolytica* (70%, 81 out of 133 samples) (Fig. 4). Also, a difference in prevalence was observed between BRD and healthy animals for *M. haemolytica* (81 and 61%, respectively), and *M. bovis* (74 and 50.7%, respectively) (Fig. 4). As a comparative analysis, the prevalence of the four BRD pathobionts using the 16S rRNA gene sequencing data was determined (see Additional File 1: Tables S3-S4). In this study, some ASVs were specifically assigned to the BRD-associated species: one ASV was assigned to *P. multocida* and one to *M. haemolytica*, five ASVs were identified as *M. bovis* in the study, and none as *H. somni* (see Additional File 1: Table S3). Based on the 16S rRNA gene data regardless of the health status, similar to the qPCR data, the prevalence of *P. multocida* was high, 97%, 127 out of 131 samples. The *M. haemolytica* ASV was only observed in 1 sample out of 131, while *Mannheimia* as a genus had a 61% prevalence and was slightly higher in BRD animals than healthy animals (see Additional File 1: Table S4). Similar to the qPCR results, the most prevalent *M. bovis* ASV, ASV13707, had a higher prevalence in BRD animals (65%) than healthy animals (41%). An ASV assigned to the genus *Histophilus* had a prevalence of 76% overall with only a small difference between BRD and healthy animals. *H. somni* is the only recognized species in the *Histophilus* genus; thus, it can be assumed the identified ASV is *H. somni* [41] (see Additional File 1: Table S3-S4).

Interestingly, the total bacterial abundance was significantly higher in the BRD animals than healthy animals; 8.49 ± 0.126 log_10_ mean copies in BRD animals and 7.99 ± 0.113 log_10_ copies in healthy animals (F_1,124.05_ = 9.5567, *p* < 0.002).

**Fig. 5:**
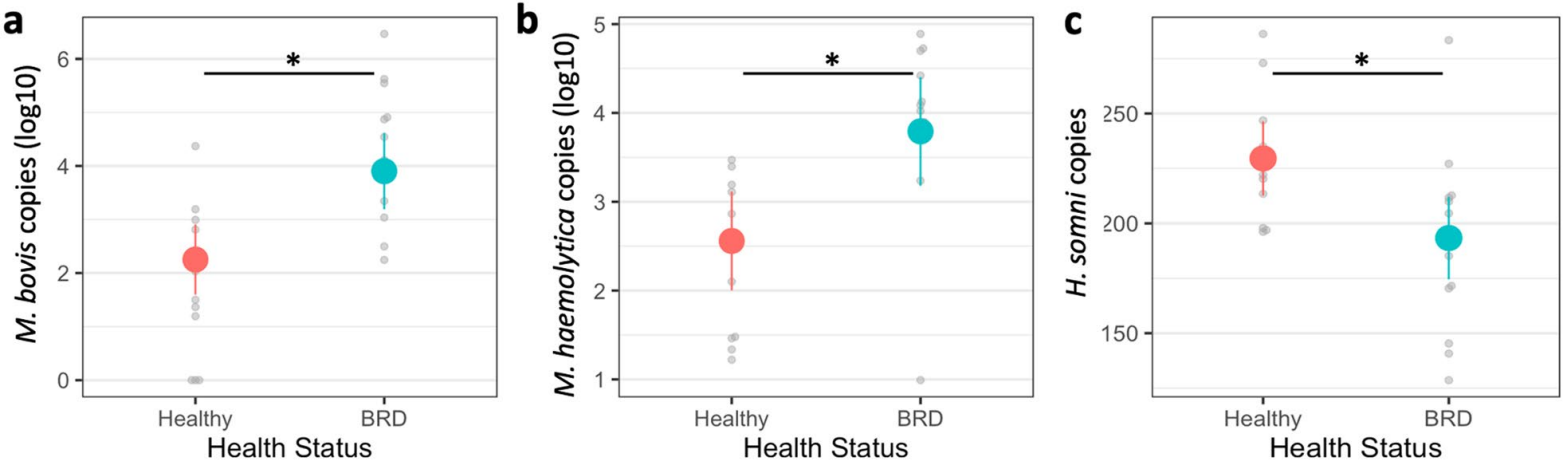
Difference in bacterial abundance per sample (200 µl of extracted DNA) for *Mycoplasma bovis* (**a)**, *Mannheimia haemolytica* (**b)** and *Histophilus somni* (**c**) between animals with (BRD) and without (Healthy) BRD clinical signs (n = 133). An asterisk (*) and horizontal line represent a statistical difference (*p* ≤ 0.05) between the two groups. Colored circles represent the means of the BRD, and healthy group, vertical lines indicate the standard error of the means, and the gray dots represent individual samples of each group

From all the animals (n = 133), the abundance per nasal sample of both *M. bovis* (F_1,122.72_ = 15.7327, *p* < 0.0001; Fig. 5a) and *M. haemolytica* (F_1,123.25_ = 10.9789, *p* < 0.0001; Fig. 5b) was increased nearly ten fold in BRD animals compared to healthy animals. On the contrary, *H. somni* abundance was about 10% lower in BRD animals (F_1,124.00_ = 8.9821, *p* < 0.003; Fig. 5c). There was no significant difference in *P. multocida* abundance based on health status. In addition, after quantifying the relative abundance of the four pathobionts in the whole bacterial community determined by the 16S rRNA gene abundance, only the relative abundance of *P. multocida* was significantly different, being higher in healthy (88.1%) than BRD animals (33.3%) animals (F_1,122.07_ = 4.1703, *p* < 0.043).

### Classification of healthy and BRD animals based on the nasal microbiome

**Fig. 6:**
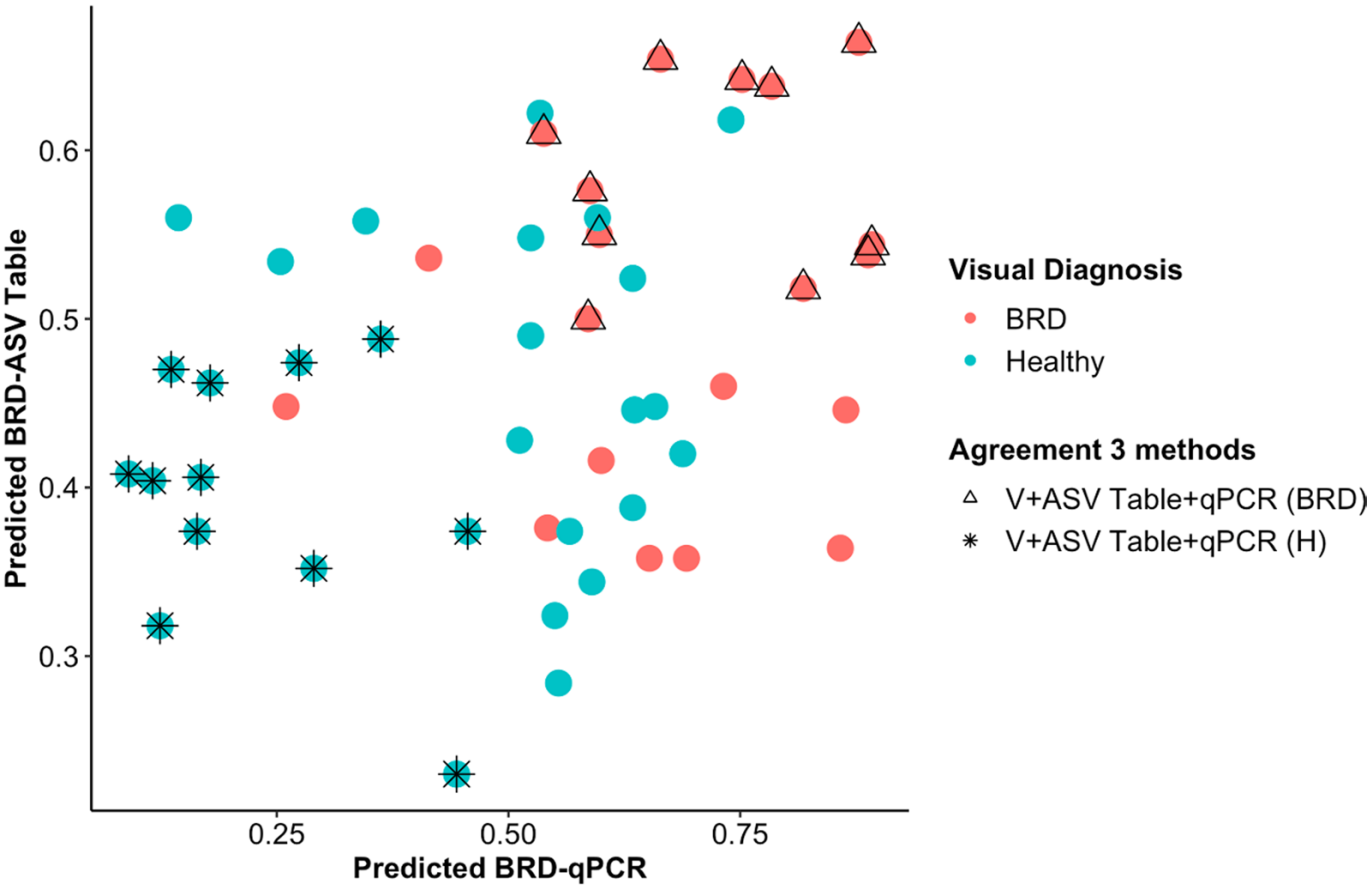
Probability of classifying animals as BRD or healthy (< 0.5 = healthy, > 0.5 = BRD) using Random Forest analysis. Classification of the animals was based on the microbial community composition (ASV table) and quantification of BRD pathobionts, 16S rRNA gene abundance and age (qPCR). The color indicates the initial animal classification based on the BRD clinical signs. Shape indicates if the animal classification agreed between the three methods: visual classification based on BRD clinical signs (V), microbial community composition (ASV Table) and quantification of BRD pathobionts and 16S rRNA gene abundance (qPCR)

Random forest analyses were performed to predict the health status of the animals based on (1) BRD pathobiont gene copy number, 16S rRNA gene copy number and animal age, or (2) the microbial community composition based on 16S rRNA amplicon sequencing. In this process, 60% of the samples (n = 124) were used for training the random forest model (n = 74) and 40% were used in the testing set (n = 50). The predicted health status was then compared to the visual observations of BRD clinical signs. In the testing set, 30 animals were identified based on the BRD clinical signs as healthy and 20 as BRD. After performing the random forest analysis using the community composition (pruned ASV table), in the testing set, 30 animals were predicted to be healthy and 20 animals were predicted as BRD, with a sensitivity of 60%, specificity of 73% and accuracy of 68%. When the BRD-pathobiont gene copy numbers, 16S rRNA gene copy number and animal age were utilized for the classification by random forests, in the testing set, a total of 17 animals were predicted as healthy and 33 animals were predicted as BRD with a sensitivity of 55%, specificity of 88% and misclassification rate of 34%. In addition, 11 out of 20 cattle visually identified as BRD-affected and 12 out of 30 visually identified as healthy agreed in the classification-based community composition (ASV Table) and quantification of BRD pathobionts and 16S rRNA gene abundance (Fig. 6). In addition, with random forest it was possible to identify the factors that have a higher impact on the classification of the samples based on their mean decrease accuracy value. When classifying the samples based on the BRD pathobionts gene copy, 16S rRNA gene copy number and animal age, it was the decrease in *H. somni* and increase in *M. haemolytica* that had the highest impact on the animal classification (mean decrease accuracy 0.023 and 0.014, respectively) (see Additional File 1: Fig. S5). Also, when classifying the samples based on the community composition predicted by 16S rRNA gene sequencing (ASV table), the ASVs with the highest impact in the classification (mean decrease accuracy > 0.01) were *Bibersteinia* spp, uncultured *Rikenellaceae* RC9 gut group, *Streptococcus* species, uncultured *Ruminococcaceae*, *Fastidiosipila*, *Azoarcus*, unclassified *Moraxellaceae*, *Ornithinimicrobium*, and unclassified *Muribaculaceae* (see Additional File 1: Fig. S5). These results bring more information regarding other possible nasal microbes that could be associated with BRD and could be used as possible markers when diagnosing BRD using the nasal microbiome.

### Nasal microbiome co-occurrence analysis

In the study, a total of 18,010 ASVs were identified in the samples. After removing rare ASVs with an average relative abundance ≤ 0.0001, 1236 ASVs remained in the analysis and were used in the co-occurrence analysis (Table 1). Positive associations indicate the ASV pairs that are more likely to co-exist in the same sample, whereas the negative associations represent the ASVs that are likely to not co-exist in the same sample.

**Table 1:** Co-occurrence analysis summary for BRD (n = 57) and Healthy (n = 74) groups

Group	ASV pair associations	ASV pair associations not included	ASV pairs remained	ASV pair associations (+)	ASV pair associations (−)	Random unclassified ASV pair combinations
BRD	763,230	60,569	702,661	92,500	15,313	594,848
Healthy	763,230	41,783	721,447	147,864	15,177	558,406

After applying the filtering thresholds to identify ASV pairs most common in the nasal cavity (ASV pairs present in more than 60% of the samples and a probability greater than 0.9), a total of 280 positive ASV pairs associations remained in the healthy group and 90 positive ASV pairs associations in the BRD group (see Additional File 1: Figs. S6 and S7). In the healthy animals, 32 ASV pairs were observed in nearly every sample (73 out of 74 healthy animals) and with a co-occurrence probability greater than 0.9 (see Additional File 1: Table S5). In the BRD animals, only eight ASV pairs were observed in nearly every sample (56 out of 57 BRD animals) and with a probability greater than 0.9 (see Additional File 1: Table S6).

After applying the rules that negative associations should never co-occur in the samples with a probability of < 0.05, a total of 178 negative ASV pair associations were identified in the healthy (see Additional File 1: Fig. S8) from which only 32 negative ASV pairs associations were identified to less likely to co-exist in the samples with a probability < 0.04 (see Additional File: Table S7). A total of 49 negative associations were identified in the BRD group (see Additional File 1: Fig. S9) from which only 13 negative ASV pair associations were identified to less likely to co-exist in the samples with a probability < 0.04 (see Additional File 1: Table S8).

In addition to identifying the most prevalent ASV pair combinations in the nasal microbiome of healthy and BRD animals, ASV pair associations between commensal microbiota and BRD-pathogens were identified with more relaxed filtering criteria (probability > 0.7). This allowed us to detect how common the BRD-pathobionts are associated with other bacteria in the cattle nasal cavity. We were able to identify in the BRD group one positive pair association between *P. multocida* and *Escherichia* present in 55 samples with a chance of occurring in the same sample of 0.948. No other ASV pair associations between BRD pathobionts and commensal nasal microbiome were observed with a probability > 0.7.

## Discussion

Bovine respiratory disease (BRD) is an ongoing health and economic problem in the dairy and beef industries, and there are multiple risk factors that make animals susceptible to BRD [3, 6]. Four major pathobionts have been identified to have a relation with BRD development: *P. multocida*,* H. somni*,* M. haemolytica*, and *M. bovis* [8, 9]. In the field of BRD research, most nasal microbiome studies have focused on characterizing the respiratory microbiome using next-generation sequencing of 16S rRNA gene amplicons [2, 42] for the relative quantification of BRD pathobionts in the respiratory tract [8, 28, 43]. However, few studies combined the two approaches (16S rRNA gene amplicon sequencing and qPCR) to characterize the nasal microbiome and quantify the presence of the BRD pathobionts. In this study, nasal samples were collected from healthy and BRD-affected animals to identify a differences in their nasal microbiomes, the prevalence and abundance of four BRD pathobionts, and co-occurrences among the taxa in the nasal microbiome or between the BRD pathobionts and nasal microbiome. Based on our results, even though 16S rRNA amplicon sequencing was effective at predicting BRD and healthy animals, this method was unable to determine that BRD-associated pathogen species were differentially abundant in the nasal cavity of BRD-affected cattle compared to healthy animals. On the other hand, by using qPCR, it was possible to identify that *M. haemolytica* and *M. bovis* were more prevalent and abundant in the nasal cavity of BRD animals, as well as to effectively predict healthy and BRD animals. This study will benefit the field of BRD research, because it was possible to detect BRD pathobionts in nasal samples and determine/predict healthy and BRD animals. This is important because nasal swabs could be used as a novel secondary clinical test for signs of BRD.

### Animals with BRD clinical signs had decreased alpha diversity in their nasal microbiome

In this study, the nasal microbiome of BRD animals presented lower alpha diversity than healthy animals. The richness of the nasal microbiome in BRD animals compared to healthy animals decreased by approximately 20%. A similar trend was observed by both Timsit et al. [31] and Holman et al. [2] in which the median richness of BRD animals decreased about 50% compared to the median richness of the healthy animals. Different from our study, these two studies collected nasopharyngeal samples, not nasal swabs, but the microbial community diversity in BRD animals decreased regardless of the type of sample collected. In our study, the phylogenetic diversity of the nasal microbiome in BRD animals was decreased by approximately 11% compared to healthy animals. There is evidence that a greater phylogenetic diversity confers more stability to the ecosystem and resistance to pathogen colonization [44, 45]. Ecosystem stability is determined by the resistance, resilience, and functional redundancy that the microbial community could possess [44, 46]. Thus, it could be hypothesized that the greater alpha diversity observed in healthy animals provided greater community stability, functional redundancy, or pathogen colonization resistance, compared to BRD animals. Nevertheless, more research is needed to test this hypothesis.

In addition to health status interaction with alpha diversity, alpha diversity was positively correlated with the environmental temperature. It has been identified that temperature can be considered an environmental condition that enhances bacterial growth, shaping the community [47, 48]. Additionally, in a longitudinal study characterizing the nasal microbiome change in human anterior nares throughout the different seasons in the year, it was identified that the bacterial community clustered based on when the samples were collected [49]. In that study, a change in the community structure (beta diversity) was observed from February to March 2010, marking the progression from winter to spring in which the ambient temperature increases progressively; unfortunately, no alpha diversity was analyzed in that study. However, it is possible that in our study, temperature could have contributed to the low alpha diversity values in the samples collected towards the end of the study, which marks a change from summer to winter 2020. Unfortunately, no other study has looked at the effect of ambient temperature on the bovine nasal microbiome as performed in our study.

### *Mycoplasma* spp. and other associated bacteria were associated with BRD animals

Taxonomical composition of the nasal samples collected from BRD, and healthy animals were mostly composed at the phylum level of *Proteobacteria*,* Firmicutes*,* Bacteroidetes*, and *Actinobacteria* regardless of the health status and agrees with previous studies where they had been identified as common nasal microbiota members regardless of the health status [50]. However, differences at the genus level were observed between BRD and healthy animals. The nasal microbial community in BRD animals had a decrease in the relative abundance of ASVs classified as *Clostridium *sensu stricto 1, unclassified *Moraxellaceae*, *Mycoplasma bovirhinis* and *Moraxella boevrei* DSM 14,165*.* In a previous study analyzing the microbial community of different sites in the upper and lower respiratory of healthy cattle, the presence of *Moraxella*,* Mannheimia*,* Clostridium senso stricto* 1, and *Mycoplasma* were identified as commensal members of the cattle nasal microbiome [50]. Our results showed that the relative abundance of *Mycoplasma* spp., *Trueperella pyogenes*, and the genera *Bibersteinia*,* Streptococcus* and *Moraxella* were significantly increased in BRD animals. A different study identified that the genera *Mycoplasma* spp., *Psychrobacter* spp. and *Mannheimia* spp. were enriched in nasal swabs collected from BRD animals compared to healthy animals [51]. Also, *T. pyogenes* and *Bibersteinia* have been identified as secondary pathogens and related with BRD cases [8, 39, 52, 53]. These results suggest that *T. pyogenes* and *Bibersteinia* in the nasal cavity could be related to the development of BRD and could be used in the process of BRD classification.

### Increased abundance of *M. haemolytica*, *M. bovis* and total bacteria in the nasal cavity of BRD animals could be an indicator of BRD clinical signs

Characterization of the nasal microbiome community with 16S rRNA gene sequencing provides knowledge of the community structure present in animals differentiated as BRD compared to healthy animals. Nonetheless, this method has low phylogenetic power to identify specific species and poorly discriminates some genera [54]. To overcome these limitations, qPCR was used to quantify *P. multocida*,* H. somni*,* M. haemolytica*, *M. bovis*, and total bacteria between BRD and healthy animals.

The total 16S rRNA bacterial gene abundance was statistically increased in BRD animals compared to healthy animals. This could be an indication that the bacterial concentration in the nasal cavity of BRD animals is higher than in healthy animals, or that a larger mucosal sample was collected from sick animals. Regarding the bacteria targeted, *P. multocida* and,* H. somni* were present in nearly all the samples regardless of health status. In a study in which nasal swabs were taken from only healthy animals, qPCR analysis showed that *P. multocida* and H. *somni* were the bacteria with the highest prevalence whereas *M. haemolytica* was the least prevalent [28]*.* While *P. multocida* and *H. somni* in the lung are considered BRD pathobionts, they could be part of the core commensal nasal microbiome [50]. Previously, *P. multocida*, has been linked with respiratory disease in dairy calves and with shipping fever in cattle [55, 56] while *H. somni* has been isolated from 10% of the lungs of animals that died of BRD, especially in association with viruses [57, 58].

Another finding in the present study is that *M. haemolytica* and *M. bovis* presented higher prevalence and abundance in nasal swabs from animals with BRD clinical signs than in apparently healthy animals, indicating possible association with the disease. Both bacterial species are recognized as commensal members of the nasal microbiome, and in situations where the host defense is compromised by stress they can access and colonize the lung [59, 60]. Furthermore, *M. bovis* has been identified as a primary pathogen for BRD and it has also been identified in deep nasopharyngeal and transtracheal aspiration samples of BRD-affected animals [34, 39]. In addition, *M. haemolytica*, has been identified in nasopharyngeal swabs and lung tissue samples of animals that died of acute fibrinous pneumonia and it also showed a coinfection with *M. bovis*, and *Mannheimia* species [8]. With these results, it is evident that *M. bovis* and *M. haemolytica* play a role in BRD development; unfortunately, the majority of these studies did not collect nasal samples to identify the presence of the BRD pathobionts in both the lung and the nasal cavity. In our study, since both *M. bovis* and *M. haemolytica* were enriched in the sick animals, it is possible that they both infected the lung, but this hypothesis would need to be tested in future studies to determine the predictive capacity of the nasal microbiome toward the lung microbiome.

A limitation in the treatment of BRD is the low accuracy in diagnosing sick animals and rapidly being able to determine the pathogen causing the disease. It is known that sick cattle will not present common clinical signs because they exhibit prey behavior in which they mask early symptoms; therefore, visual symptoms are often insufficient to identify sick animals [61, 62]. Comparison of the nasal microbial community composition and BRD-associated pathogen quantification agreed at about 70% in classifying healthy and BRD animals when compared to the visual classification based on BRD clinical signs. In this study, one animal that was visually classified as BRD, but was classified as healthy by the two microbiological methods and based on the qPCR data (< 0.5 probability of being classified as BRD), had high *H. somni*, and low *M. haemolytica* abundance similar to the animals correctly classified as healthy by the three methods. A different animal that was visually identified as healthy but classified as BRD by the two methods (> 0.6 probability of being classified as BRD animals), had low *H. somni* with high *M. haemolytica* similar to the animals classified as BRD by the three methods*.* Thus, a combination of these methods could potentially be used in the future to increase the accuracy of diagnosing BRD.

### BRD animals had decreased bacterial co-occurrence in the nasal cavity compared to healthy animals

Bacterial-bacterial interactions can be synergistic or antagonist, and depending on the signal detected, can change their behavior on a population-wide scale [63]. It is thought that commensal members of the nasal microbiome could play a role in the health of the host by decreasing or enhancing the chance of pathogenic bacterial colonization [33, 34]. Even though most previous BRD studies compared the nasal microbial community between healthy and BRD-affected animals, few studies have investigated bacterial interactions or associations in the nasal microbiome. Thus, a co-occurrence analysis was performed to identify any ASV pairs specific to the BRD and healthy groups.

In this study, the nasal microbiome of healthy animals had more positive associations (ASVs pairs that co-exist in the same sample) and negative associations (ASVs pairs that do not co-exist in the same sample) than the BRD animals. It has been shown that microbes that coexist in close physical proximity to each other, display increased likelihood to interact with each other [64]. Thus, the difference in the number of co-occurrence taxa could be attributed to the higher diversity observed in the healthy compared to those affected by BRD. Nonetheless, more research is needed to determine the type of co-occurrence or interaction present in the nasal cavity of BRD and healthy animals (e.g., commensalism, synergism, competition, amensalism, and predation) [64] that could provide more information regarding how BRD develops. Fewer bacterial associations observed in the BRD group might indicate microbial dysbiosis in the BRD animals [65]. However, a previous study performed in human respiratory microbiome indicated that microbial dysbiosis could not be determined as the cause of the disease [66].

Only one previous study investigated microbial co-occurrence patterns present in BRD and healthy animals. In this study, the bacterial genera *Mannheimia* and *Histophilus* were substantially increased in BRD animals and the genus *Fusobacterium* co-occurred with *Caviibacter* in BRD-affected animals [65]. Also, negative associations between *Moraxella*,* Corynebacterium*, and *Pasteurella* and the archaeal genus *Methanobrevibacter*, with *Mycoplasma* were observed in nasopharyngeal samples of cattle transported to the feedlot [67]. Nonetheless, in our study, we found positive associations between *M. bovis* and *Corynebacterium*, albeit with a low probability of 0.1 and 0.2 in the healthy animals and with a probability of 0.3 in BRD animals. No other associations reported previously [67] were observed in the current study and could indicate that BRD pathobionts are less likely to co-occur with other members of the cattle nasal microbiome. Another interesting result in this study is that in healthy animals, *Acinetobacter*,* Methanobrevibacter*, and *Corynebacterium* 1 were the genera with the most co-occurring interactions with other members of the nasal microbiome (probability > 0.9), and these three bacteria had been identified as members of the cattle nasal microbiome [42, 67, 68]. However, the genus *Acinetobacter* has been found to be increased in BRD-affected cattle [65]. Thus, the role of *Acinetobacter* in the nasal microbiome remains unclear. In the case of bacterial associations in the BRD animals, bacteria genera *Flavobacterium*,* Clostridium *sensu stricto* 1*,* Bacteroides* and the archaeal genus *Methanobrevibacter* were the most likely to be associated with nasal microbiome members and again, these bacteria have been identified as members of the core nasal microbiome in cattle [50].

Despite the strong bacterial associations within the commensal bacteria, no BRD pathobionts were identified to have common associations with members of the nasal microbiome. It has been found that bacteria can enhance or prevent the growth of other bacteria when present in the same environment [64]. An in vitro study demonstrated that the presence of *Staphylococcus epidermidis*,* Rhodococcus* spp., *Moraxella* spp., *Corynebacterium* spp., *Micrococcus*, and *Streptococcus viridans* enhanced the growth of *P. multocida*,* H. somni*, and *M. haemolytica* [33]; however, no other studies have investigated bacterial interactions in the nasal cavity of healthy and BRD animals. Thus, it is possible that members of the nasal microbiome community are not enhancing or interacting with BRD-associated members in vivo. This could give rise to a new working hypothesis that in the nasal microbiome BRD pathobionts have a neutralism interaction, meaning that BRD pathobionts can be present in the same environment but never interact with other commensal bacteria [64]. Lastly, another explanation for these results could be that BRD pathobionts produce metabolites that decrease the ability of other commensal members to co-exist in the same environment and co-occurrence interactions would therefore not be observable. Some studies have looked at virulence factors of *P. multocida*,* H. somni*,* M. haemolytica*, and *M. bovis* [55, 60, 69], but these factors affect the host immune response; unfortunately, no studies have investigated how the presence of these pathogens and generation of metabolites could affect the growth or presence of the nasal microbiome in cattle.

## Conclusions

Bacterial 16S rRNA gene sequencing demonstrated that the nasal microbiome of BRD animals have lower alpha diversity values (richness and evenness) than healthy animals. Lower alpha diversity could decrease microbial community stability in BRD animals, possibly making them more susceptible to pathogen colonization. In addition to decreased diversity, animals in the BRD group had fewer bacterial cooccurrences than healthy animals potentially indicating dysbiosis in the nasal microbial community of animals with BRD clinical signs. Co-occurrence analysis demonstrated that core members of the cattle nasal microbiome are the most common bacteria to co-occur with other members of the nasal microbiome, while BRD pathobionts co-occurred with few members of the cattle nasal microbiome. Detection and quantification of BRD pathobionts revealed that *H. somni* and *P. multocida* are commonly present in the nasal microbiome regardless of health status. However, the prevalence and abundance of *M. haemolytica* and *M. bovis* were increased in the BRD group, demonstrating a possible connection between these two bacterial species and BRD development. In addition, microbial community analysis indicated that increased relative abundance of *Mycoplasma* spp.,* Truperella pyogenes*,* Bibersteinia* spp. in the nasal cavity of BRD animals could be related with BRD development. Characterization of the nasal microbial community composition, quantification of BRD pathobionts and the total bacterial 16S rRNA gene were largely in agreement in classifying the animals as BRD or healthy in comparison with classifying the animals based on the visual clinical signs. Thus, these marker microorganisms in nasal samples could be used as a potential secondary clinical method to provide additional information about animals after being identified by the visual clinical signs as needing additional clinical evaluation.

## Materials

### Animal population and selection

All procedures involving animal use were approved by the Purdue University Animal Care and Use Committee (protocol #1906001911). A total of 133 Holstein steers approximately 6–7 months old, housed in the same environment at a feedlot (Indiana, USA) were sampled from July to December 2020; the animals selected were only sampled once. Animals with BRD clinical signs were identified following the DART method that uses depression, appetite loss, respiratory character change and rectal temperature [38] as clinical signs of disease. Animals that exhibited 2 out of 3 clinical signs (not including rectal temperature) were selected and are referred to as BRD animals. Once an animal was identified to have BRD clinical signs, one or two apparently healthy pen mates were also selected for nasal swab sampling. More healthy animals were selected than BRD animals in case one of the healthy animals later displayed BRD clinical signs after sample collection. Healthy animals were identified as animals that did not present any BRD clinical signs such as depression, respiratory character change and appetite loss. After selecting the BRD and healthy pen-mates, rectal temperature was measured to corroborate the visual classification, a temperature > 103 °F was established to corroborate BRD animals. Nonetheless, measurement of rectal temperature did not always indicate sickness, as a few animals without clinical signs also presented rectal temperature > 103 °F. The animals selected for the study were not previously treated (individually or mass-mediated) for BRD or any other disorder with antibiotics for the previous 100 days. After the end of the study, animal health records were reviewed and any healthy animals that were treated for BRD after sampling were removed from the study. A total of 75 healthy and 58 BRD animals were included in the study.

### Cattle nasal swab collection and DNA extraction

After identifying healthy and BRD animals, the outside of the nostrils were cleaned with a paper towel, and two unguarded swabs were inserted simultaneously about 3–5 cm deep into one nostril and then into the other nostril, rotating against the mucosal surfaces for about 5 s in each nostril. Nasal swabs were placed in an empty 15 mL conical tube; the tubes were labeled and transported (1.5 h on ice) to the laboratory for processing. During sampling, rectal temperature, and pen ID were collected for each animal for further data analysis. Nasal swabs were processed to extract the bacterial and mucosa content from the tip of the swabs by adding 1 mL of nuclease-free water and then mixed horizontally by vortex for 5 min, after which the tip of the swab was removed and the liquid was centrifuged (6000 × for 10 min) to form a pellet of the swab contents. The supernatant was removed and the mucosal pellet was stored at − 20 C at this point. Total DNA was extracted from the pellets using the DNeasy Blood & Tissue Kit (Qiagen, Germantown, MD, USA) following the method described in Holman et al. [2]. 16S rRNA library pool preparation were performed following the Kozich et al. [70] protocol. The amplicons were sequenced using an Illumina MiSeq Sequencer (2 × 250 paired-end) at the Purdue Genomic Core Facility.

Raw sequence data obtained from the 16S rRNA gene amplification were analyzed using Quantitative Insight Into Microbial Ecology (QIIME2) v.2020.2. Using the DADA2 [71] denoising step, the forward and reverse sequences were trimmed at position 0, and the forward and reverse sequences were truncated at position 251 and 223, respectively, to obtain sequences with a quality > Q30. The forward and reverse reads were then merged. The taxonomy of each sequence was assigned using SILVA 132, 515F/806 region database. Then, the sequences were rarified to 40,420 sequences per sample to calculate the alpha and beta microbial diversity in the nasal swab samples; in this step, seven samples (6 healthy and 1 BRD) were removed due to low sequence count. Alpha diversity was estimated in QIIME2 using the Observed OTUs and Chao1 metrics for richness, Pielou index for evenness and Faith (Faith’s PD) for phylogenetic diversity [72–75]. The Bray–Curtis Dissimilarity Index and Weighted UniFrac were used to analyze the Beta diversity [76] and plotted as principal coordinate analysis (PCoA) using R v. 4.0.3

A negative binomial distribution method, DESeq2 [77], was used to determine differentially abundant taxa between BRD and healthy animals. The unrarefied ASV table was used as the input for the analysis (18,010 ASVs) ASVs with a log_2_ fold change > 2 and statistical significance of *p* ≤ 0.05 were selected as differentially abundant ASV between BRD and healthy animals.

### Mock community and empty swabs sequencing analysis

The raw 16S rRNA sequences from the mock community of 20 known bacterial strains DNA (20 Strain Even Mix 138 Genomic Material; ATCC® MSA-1002TM), and the DNA extraction negative controls used as DNA extraction negative control were analyzed separately using QIIME2 v.2020.2. as described above. To evaluate the mock community sequencing quality, we used the QIIME2 (v.2-2020.2) function evaluate_seqs [78]. The presence of contaminants during DNA extraction and sequencing was determined by comparing the mock community (positive control) taxonomical composition to the mock community known composition. Any ASVs in the mock community (positive control) that did not match mock community reference sequences and were also present in the DNA extraction negative controls at a relative concentration higher than 10% were considered contaminants and were removed from the data.

### qPCR analysis for BRD pathobionts and 16S rRNA gene

DNA extracted from pure isolates of *P. multocida*,* H. somni*, and *M. haemolytica* acquired from the Indiana Animal Disease Diagnostic Laboratory (ADDL) at Purdue University and DNA from *M. bovis* strain 25,523 (American Type Culture Collection) was used to generate the qPCR standard curve to quantify the abundance of BRD pathobionts. PCR assays were performed to target the genes mentioned in Table 2 [28, 79].

**Table 2:** BRD pathobiont primers and probes sequences for PCR and qPCR reactions

Target	Target gene	Primer name	Sequence (5′–3′)	Size (bp)	Ref
*M. haemolytica*	*sod*A	Mh-SGF	AGCAGCGACTACTCGTGTTGGTTCAG	26	[80]
*M. haemolytica*	*sod*A	Mh-SGR	AAGACTAAAATCGGATAGCCTGAAACGCCTG	31	[80]
*M. haemolytica*	*sod*A	Mh-BV1P*	TTCAACCGCTAACCAGGACAACCCAC	26	[80]
*P. multocida*	16S rRNA	Pm-TMF	CGCAGGCAATGAATTCTCTTC	21	[81]
*P. multocida*	16S rRNA	Pm-TMR	GGCGCTCTTCAGCTGTTTTT	20	[81]
*P. multocida*	16S rRNA	Pm-TMP*	ACTGCACCAACAAATGCTTGCTGAGTTAGC	30	[81]
*H. somni*	16S rRNA	Hs-TMF	AGGAAGGCGATTAGTTTAAGAGATTAATT	29	[81]
*H. somni*	16S rRNA	Hs-TMR	TCACACCTCACTTAAGTCACCACCT	25	[81]
*H. somni*	16S rRNA	Hs-TMP*	ATTGACGATAATCACAGAAGAAGCACCGGC	30	[81]
*M. bovis*	*opp*D	PMB996-F	TCAAGGAACCCCACCAGAT	19	[79]
*M. bovis*	*opp*D	PMB1066-R	AGGCAAAGTCATTTCTAGGTGCAA	24	[79]
*M. bovis*	*opp*D	Mbovis1016*	TGGCAAACTTACCTATCGGTGACCCT	26	[79]

*Probe fluorophore and double quencher: 5′ 6-FAM/ZEN/3′ IBFQ

The PCR assays for *P. multocida*, *H. somni*, *M. haemolytica*, and *M. bovis* were performed in a 50 μl volume consisting of 25 μl of iTaq™ Universal Probes Supermix (BioRad, CA, USA), 12.5 μl Primer/Probe mix (Integrated DNA Technologies IDT, Coralville, Iowa, USA) listed in Table 1 with a concentration of 300 nM for the primers and 100 nM for the probes as reported in Thomas et al. [28], 10 μl nuclease-free water and 2.5 μl of nucleic acid template. PCR assays were performed in an Eppendorf Mastercycler Gradient Model 533 and the cycling conditions for *H. somni* and *P. multocida* were 95 °C for 3 min followed, by 40 cycles of 95 °C for 3 s and 60 °C for 60 s. Cycling conditions for *M. haemolytica* were as follows: 95 °C for 3 min, followed by 40 cycles of 95 °C for 3 s and 69 °C for 60 s. Cycling conditions for *M. bovis* were 95 °C for 10 min, followed by 45 cycles of 95 °C for 30 s, and 60 °C for 60 s.

PCR-grade water was used as a negative control. PCR amplification quality was checked via gel electrophoresis. *P. multocida*,* H. somni*,* M. haemolytica* and *M. bovis* gene amplicons were cleaned and purified using a Monarch PCR and DNA Cleanup kit (New England BioLabs, MA, USA), amplicon concentration was determined by Qubit dsDNA HS Assay kit (Thermo Fisher Scientific, PA, USA) and the amplicons were stored at − 20 °C until further use. Amplicon copy number was calculated using the concentration and length of the amplicon.

For the qPCR standard sample dilutions, amplicon serial dilutions (10^8^ to 10^0^) were created for each BRD pathobiont. The standard curve equation was generated using a linear regression of technical triplicate average quantification cycle (Cq) and log_10_ amplicon copies/μl as known from each dilution. The qPCR technical triplicate assays were performed in 20 μl total volume containing 10 μl iTaq™ Universal Probes Supermix (BioRad, CA, USA), 5 μl Primers/Probes and 5 μl of each bacterium amplicons generated in the PCR step. The qPCR assays were performed in CFX96 Real-Time System Thermal Cycler (BioRad, CA, USA). Amplification efficiency (*E*) was calculated using the slope of the standard curve and the formula: *E* (%) = (− 1 + 10^−1/slope^) × 100.

To quantify the abundance of *P. multocida*,* H. somni*,* M. haemolytica* and *M. bovis* in the nasal swabs, qPCR reactions were performed in triplicate, with a separate reaction for each bacterium using the cycling conditions and the primers and probe concentration described in the BRD pathobiont PCR step. To account for inter-plate variability only one technical replicate was included in a single plate. The BRD pathobiont copy number obtained from 5 μl of extracted DNA used in the qPCR assays were divided by 5 to get the number of copies per 1 μl and then, multiplied by the total volume obtained from the DNA extraction process (200 µl). This process determined the absolute abundance of each bacterium in the total extracted sample. In each qPCR plate, one standard curve dilution was utilized as the positive control and PCR-grade water as the negative control.

To quantify the 16S rRNA gene abundance present in the animal’s nasal cavity, DNA extracted from the nasal samples was used as the template to generate 16S rRNA amplicons by PCR. 16S rRNA gene amplification was performed in an Eppendorf Mastercycler Gradient Model 533, using the primers 8F (5′ AGAGTTTGATCCTGGCTCAG 3′) and 1492R (5′ ACGGTTACCTTGTTACGACTT 3′) to obtain the bacterial 16S rRNA gene amplicon that was further utilized as the qPCR template to generate the standard curve [82]. PCR assays were performed in a 50 μl volume reaction consisting of 42.5 μl of AccuPrime™ Pfx SuperMix (Thermo Fisher Scientific, MA, USA), 2.5 μl of each 10 μM primer (8F/1492R), 1.5 μl of Nuclease-free water and 1 μl DNA template. The primer concentration and PCR cycling conditions were performed as stated in the protocol by Kozich et al. [70] protocol. PCR-grade water was used as a negative control and as a mock community (20 Strain Even Mix 138 Genomic Material; ATCC® MSA-1002™) as a positive control. PCR was verified and amplicons purified as described above.

For the qPCR standard curve generation, a serial dilution (10^8^ to 10^0^) was made of the 16S rRNA gene amplicons. The qPCR assays were performed in 20 μl total volume containing 10 μl LightCycler 480 SYBR Green I Master (Thermo Fisher Scientific, PA, USA), 5 μl Primers/Probes and 5 μl of the gene amplicons. The qPCR assays were performed in a CFX96 Real-Time System Thermal Cycler (BioRad, CA, USA) using the universal bacteria primers 1132F and 1108R [83] at a concentration of 6 pmol with cycling conditions of 40 cycles of 95 °C for 15 s and 60 °C for 1 min. The standard curve generation, calculation of amplification efficiency, and gene abundance were performed as described in the previous section. One standard curve dilution was utilized as the positive control and PCR-grade water as the negative control in each of the qPCR plates.

The prevalence of *P. multocida*,* H. somni*,* M. haemolytica* and *M. bovis* was calculated as the number of animals that tested positive for each bacterial species from the total numbers of samples (percentage) and the prevalence was also calculated between healthy and BRD animals. Samples with a Cq value greater than the Cq obtained with the endpoint of the standard curve (see Additional File 1: Table S9) were determined to be false positives. In addition, the relative abundance of the four BRD pathobionts was determined by dividing the bacterium copy number by the 16S rRNA gene copy number obtained per sample (determined by 16S rRNA gene abundance).

### Statistical analysis for 16S rRNA gene sequencing and qPCR data

Alpha diversity metrics (Observed ASVs, Chao1, Pielou, and Faith’s PD) and quantification of BRD-associated pathogen gene copy number and 16S rRNA gene abundance (qPCR) were analyzed using a General Linear Mixed Model with the random factor specified to only include random slopes using the afex package [84]. Status of clinical signs (BRD or Healthy) was included as a fixed factor and pen as a random factor. The age and date of sampling were included as continuous factors in the model. We checked assumptions of normality of the residuals and homogeneity of variance were checked using the afex package. We log-transformed the dependent variables when the assumptions were not met. The F values reported include the listing of the degrees of freedom associated with the model. To test the difference in beta diversity between healthy and BRD animals, a permutational multivariate analysis of variance test (PERMANOVA) of the Bray–Curtis dissimilates and Weighted UniFrac distances was performed using the adonis function from the vegan package [85]. In addition, a dispersion test was performed to determine the distance of the samples from the centroids of the two groups (BRD or healthy animals) using the vegan package [85], followed by a permutation test of multivariate homogeneity of groups dispersion using the vegan package [85]. Statistical significance was defined as *p* ≤ 0.05. All the statistical analyses involving the microbiome composition were performed in R v. 4.0.3 and the code, and data used in this study are available at https://github.com/EuniceCenteno/BRDNasal.

### Classification of healthy and BRD animals using random forest

A random forest analysis was performed with the rarified ASV table using the randomForest package in R v. 4.0.3. First, the samples (n = 124) were randomly divided into training set (60% of the data, n = 74) and testing set (40% of the samples, n = 50). ASV table obtained from the 16S rRNA gene sequencing was pruned to only include the ASVs with a the relative abundance greater than 0.0001 with 40,420 reads or more per sample leaving 1257 ASVs for the analysis. Random forest analysis was then performed separately using the microbial community (pruned ASV table) or BRD-pathobiont abundance (qPCR result) with 16S rRNA gene abundance (qPCR result) and animal age. To determine the classification accuracy, the sensitivity (Eq. 1), specificity (Eq. 2) and misclassification rate were calculated. Misclassification rate was calculated by comparing the animal visual classification with the classification assigned to the sample using Random Forest. Also, the samples classified as BRD or healthy based on the random forest that agreed with the visual classification were identified. Lastly, the factors that had the highest impact in classifying the samples were identified based on the mean decrease accuracy > 0.001.

1 $$Sensitivity =\frac{\# \;of\; true\; positives}{\# \;of\; true\; positives + \#\; of\; false \;negatives}$$

2 $$Specificity = \frac{\# \; of\, true\, negatives}{\#\; of \;true \;negatives + \# \;of\; false \;positives}$$

### Microbial community co-occurrence analysis

A co-occurrence analysis was performed to identify ASV pairs that coexist in the sample (positive associations) or do not co-exist (negative association) in the nasal cavity of healthy and BRD animals. The ASV table was subset into two tables: samples from BRD and healthy animals. ASVs with an average relative abundance < 0.0001 were removed from the dataset, and 40,420 sequence counts or more per sample were used in the analysis. A total of 1236 ASVs were used in the analysis. Then, the ASV table was converted to a binary format: presence (1) and absence (0). Co-occurrence analysis was performed with the package cooccur [86] in R (v. 4.0.3). Co-occurrence results were filtered to require that positive association ASV pairs be significantly different (*p* ≤ 0.05), present in at least 60% of the total number of sample (BRD n > 34 samples and Healthy n > 45 samples), and with a probability > 0.9 that the two ASVs will co-occur in the same sample. It was required that negative associations have a significance of *p* ≤ 0.05, with probability < 0.05 that the two ASVs would occur in the same sample and never co-occur in the samples.

Once the ASV pair combinations were identified, any pair combination (positive or negatively associated) with the BRD-pathobionts were selected with a probability threshold of 0.7. This allowed us to observe the association of the pathogens with any other bacteria present in the nasal cavity of healthy and BRD animals.

## Supplementary Information

**Additional File 1.** Supplementary Results. **Figure S1. **Top 10 most abundant genera present in the DNA extraction negative controls used as a negative control in the DNA extraction and sequencing step. **Figure S2. **Holstein steer nasal microbiome alpha diversity relative to the date of collection. **Figure S3. **Variation in the cattle’s nasal alpha diversity estimated relative to the average daily temperature (°F). **Figure S4.** Average relative abundance of the 10 most abundant phyla (**a**), family (**b**), and genera (**c**) present in the nasal microbiome of BRD and healthy animals. **Figure S5. **Variable importance measured by random forest indicated by the mean decrease accuracy. **Figure S6. **Visual representation of the positive ASV co-occurrence analysis in the healthy group (n = 74) with a probability of occurrence in the same sample > 0.9. **Figure S7. **Visual representation of the positive ASV co-occurrence analysis in the BRD group (n = 57) with a probability of occurrence in the same sample > 0.9. **Figure S8. **Visual representation of the negative ASV co-occurrence analysis of the healthy group (n = 74) with a probability of occurrence in the same sample < 0.05. **Figure S9. **Visual representation of the negative ASV co-occurrence analysis in the BRD group (n = 57) with a probability of occurrence in the same sample < 0.05. **Table S1. **Variation in the cattle’s nasal alpha diversity relative to the animal age (months) analyzed using General Linear Mixed Model. **Table S2. **Sample average distance to the centroids of the BRD and healthy groups. **Table S3. **Prevalence of ASVs assigned as* P. multocida*,* H. somni* and* M. bovis* the nasal cavity of BRD (n = 75) and healthy (n = 74) groups based on 16S rRNA gene sequencing.  **Table S4.** Prevalence of the genera* Pasteurella*,* Histophilus*,* Mannheimia* or* Mycoplasma* in the nasal cavity of Holstein steers (n=131) and between BRD (n = 75) and healthy (n = 74) animals based on 16S rRNA gene sequencing. **Table S5.** Positive ASV pair combinations present in 73 out of 74 healthy samples with a probability of occurrence in the same sample > 0.9. **Table S6.** Co-occurrence analysis with positive ASV pair combinations present in 56 out of 57 BRD samples with a probability of occurrence in the same sample of 1. **Table S7**. Co-occurrence analysis showing negative ASV pair combinations that are not likely to co-exist in the heathy group with a probability < 0.04.  **Table S8.** Co-occurrence analysis showing negative ASV pair combinations that are not likely to co-exist in the BRD group with a probability < 0.04. **Table S9.** Evaluation of *Pasteurella multocida, Histophilus somni, Mannheimia haemolytica* and *Mycoplasma bovis* using qPCR assays.

## Data Availability

Sequences were deposited in the NCBI sequence read archive (SRA) database under Bioproject PRJNA746809, BioSamples SAMN20244099–SAMN20244251. Additional files used in data analysis for this study are available at https://github.com/EuniceCenteno/BRDNasal for reference and reproducibility.

## Abbreviations

BRD: Bovine respiratory disease
qPCR: Quantitative PCR
PCR: Polymerase chain reaction
ASVs: Amplicon sequence variants
LRT: Lower respiratory tract
URT: Upper respiratory tract
QIIME: Quantitative Insight Into Microbial Ecology
PCoA: Principal coordinate analysis
ADDL: Indiana Animal Disease Diagnostic Laboratory
Cq: Quantification cycle
*E*: Amplification efficiency
PERMANOVA: Permutational multivariate analysis of variance test
BRSV: Bovine respiratory syncytial virus
BVDV: Bovine viral diarrhea virus
s: Seconds

## References

[1] Babcock AH, Renter DG, White BJ, Dubnicka SR, Scott HM (2010). Temporal distributions of respiratory disease events within cohorts of feedlot cattle and associations with cattle health and performance indices. Prev Vet Med.

[2] Holman DB, McAllister TA, Topp E, Wright ADG, Alexander TW (2015). The nasopharyngeal microbiota of feedlot cattle that develop Bovine Respiratory Disease. Vet Microbiol.

[3] Chirase NK, Greene LW (2000). Influence of oral natural interferon-alpha on performance and rectal temperature of newly received beef steers.

[4] Vogel GJ, Bokenkroger CD, Rutten-Ramos SC, Bargen JL (2015). A retrospective evaluation of animal mortality in US feedlots: rate, timing, and cause of death. Bov Pract..

[5] Edward AJ (1996). Respiratory disease in feedlot cattle in the central USA. Bov Pract.

[6] Snowder GD, Van Vleck LD, Cundiff LV, Bennett GL (2006). Bovine respiratory disease in feedlot cattle: environmental, genetic, and economic factors. J Anim Sci.

[7] Bowland SL, Shewen PE (2000). Bovine respiratory disease: commercial vaccines currently available in Canada. Can Vet J.

[8] Klima CL, Zaheer R, Cook SR, Booker CW, Hendrick S, Alexander TW (2014). Pathogens of Bovine Respiratory Disease in North American feedlots conferring multidrug resistance via integrative conjugative elements. J Clin Microbiol.

[9] Mosier D (2014). Review of BRD pathogenesis: the old and the new. Anim Health Res Rev.

[10] Ackermann MR, Derscheid R, Roth JA (2010). Innate immunology of Bovine Respiratory Disease. Vet Clin North Am Food Anim Pract.

[11] Uehara A, Fujimoto Y, Fukase K, Takada H (2007). Various human epithelial cells express functional Toll-like receptors, NOD1 and NOD2 to produce anti-microbial peptides, but not proinflammatory cytokines. Mol Immunol.

[12] Zeineldin M, Lowe J, Aldridge B (2019). Contribution of the mucosal microbiota to bovine respiratory health. Trends Microbiol.

[13] Caswell JL (2014). Failure of respiratory defenses in the pathogenesis of bacterial pneumonia of cattle. Vet Pathol.

[14] Timsit E, Holman DB, Hallewell J, Alexander TW (2016). The nasopharyngeal microbiota in feedlot cattle and its role in respiratory health. Anim Front.

[15] Loong TW (2003). Understanding sensitivity and specificity with the right side of the brain. BMJ.

[16] White BJ, Renter DG (2009). Bayesian estimation of the performance of using clinical observations and harvest lung lesions for diagnosing Bovine Respiratory Disease in post-weaned beef calves. J Vet Diagn Invest.

[17] Wolfger B, Timsit E, White BJ, Orsel K (2015). A systematic review of Bovine Respiratory Disease diagnosis focused on diagnostic confirmation, early detection, and prediction of unfavorable outcomes in feedlot cattle. Vet Clin North Am Food Anim Pract.

[18] Idoate I, Vander Ley B, Schultz L, Heller M (2015). Acute phase proteins in naturally occurring respiratory disease of feedlot cattle. Vet Immunol Immunopathol.

[19] Schaefer AL, Cook NJ, Church JS, Basarab J, Perry B, Miller C (2007). The use of infrared thermography as an early indicator of Bovine Respiratory Disease Complex in calves. Res Vet Sci.

[20] Schaefer AL, Cook NJ, Bench C, Chabot JB, Colyn J, Liu T (2012). The non-invasive and automated detection of Bovine Respiratory Disease onset in receiver calves using infrared thermography. Res Vet Sci.

[21] Ollivett TL, Caswell JL, Nydam DV, Duffield T, Leslie KE, Hewson J (2015). Thoracic ultrasonography and bronchoalveolar lavage fluid analysis in Holstein calves with subclinical lung lesions. J Vet Intern Med.

[22] Goldansaz SA, Guo AC, Sajed T, Steele MA, Plastow GS, Wishart DS (2017). Livestock metabolomics and the livestock metabolome: a systematic review. PLoS ONE.

[23] Moore RE, Kirwan J, Doherty MK, Whitfield PD (2007). Biomarker discovery in animal health and disease: the application of post-genomic technologies. Biomark Insights.

[24] Gupta S, Mortensen MS, Schjørring S, Trivedi U, Vestergaard G, Stokholm J (2019). Amplicon sequencing provides more accurate microbiome information in healthy children compared to culturing. Commun Biol.

[25] Sontakke S, Cadenas MB, Maggi RG, Diniz PPVP, Breitschwerdt EB (2009). Use of broad range 16S rDNA PCR in clinical microbiology. J Microbiol Methods.

[26] Fulton RW, Confer AW (2012). Laboratory test descriptions for Bovine Respiratory Disease diagnosis and their strengths and weaknesses: gold standards for diagnosis, do they exist?. Can Vet J.

[27] Kishimoto M, Tsuchiaka S, Rahpaya SS, Hasebe A, Otsu K, Sugimura S (2017). Development of a one-run real-time PCR detection system for pathogens associated with Bovine Respiratory Disease Complex. J Vet Med Sci.

[28] Thomas AC, Bailey M, Lee MRF, Mead A, Morales-Aza B, Reynolds R (2019). Insights into *Pasteurellaceae* carriage dynamics in the nasal passages of healthy beef calves. Sci Rep.

[29] Jeyaseelan S, Sreevatsan S, Maheswaran SK (2002). Role of *Mannheimia haemolytica* leukotoxin in the pathogenesis of Bovine Pneumonic Pasteurellosis. Anim Heal Res Rev.

[30] Singh K, Ritchey JW, Confer AW (2011). *Mannheimia haemolytica*: Bacterial-host interactions in bovine pneumonia. Vet Pathol.

[31] Pratelli A, Cirone F, Capozza P, Trotta A, Corrente M, Balestrieri A, Buonavoglia C (2021). Bovine respiratory disease in beef calves supported long transport stress: an epidemiological study and strategies for control and prevention. Res Vet Sci.

[32] Cirone F, Padalino B, Tullio D, Capozza P, Surdo ML, Lanave G, Pratelli A (2019). Prevalence of pathogens related to Bovine Respiratory Disease before and after transportation in beef steers: preliminary results. Animal.

[33] Corbeil LB, Woodward W, Ward ACS, Mickelsen WD, Paisley L (1985). Bacterial interactions in bovine respiratory and reproductive infections. J Clin Microbiol.

[34] Timsit E, Workentine M, van der Meer F, Alexander T (2018). Distinct bacterial metacommunities inhabit the upper and lower respiratory tracts of healthy feedlot cattle and those diagnosed with bronchopneumonia. Vet Microbiol.

[35] Amat S, Subramanian S, Timsit E, Alexander TW (2017). Probiotic bacteria inhibit the bovine respiratory pathogen *Mannheimia haemolytica* serotype 1 *in vitro*. Lett Appl Microbiol.

[36] Amat S, Timsit E, Baines D, Yanke J, Alexander TW (2019). Development of bacterial therapeutics against the bovine respiratory pathogen *Mannheimia haemolytica*. Appl Environ Microbiol.

[37] Timsit E, McMullen C, Amat S, Alexander TW (2020). Respiratory bacterial microbiota in cattle: from development to modulation to enhance respiratory health. Vet Clin North Am Food Anim Pract.

[38] Griffin D, Chengappa MM, Kuszak J, Mcvey DS (2010). Bacterial pathogens of the Bovine Respiratory Disease Complex. Vet Clin North Am Food Anim Pract.

[39] Pardon B, Buczinski S (2020). Bovine Respiratory Disease diagnosis what progress has been made in infectious diagnosis?. Vet Clin North Am Food Anim Pract.

[40] Zeineldin M, Lowe J, de Godoy M, Maradiaga N, Ramirez C, Ghanem M (2017). Disparity in the nasopharyngeal microbiota between healthy cattle on feed, at entry processing and with respiratory disease. Vet Microbiol.

[41] Schoch CL, Ciufo S, Domrachev M, Hotton CL, Kannan S, Khovanskaya R, Leipe D, Mcveigh R, O’Neill K, Robbertse B, Sharma S, Soussov V, Sullivan JP, Sun L, Turner S, Karsch-Mizrachi I (2020). Database.

[42] McMullen C, Orsel K, Alexander TW, van der Meer F, Plastow G, Timsit E (2019). Comparison of the nasopharyngeal bacterial microbiota of beef calves raised without the use of antimicrobials between healthy calves and those diagnosed with Bovine Respiratory Disease. Vet Microbiol.

[43] Mohan S, Pascual-Garrigos A, Brouwer H, Pillai D, Koziol J, Ault A (2021). Loop-mediated isothermal amplification for the detection of *Pasteurella multocida*, *Mannheimia haemolytica*, and *Histophilus somni* in Bovine Nasal Samples. ACS Agric Sci Technol.

[44] Cardinale BJ, Duffy JE, Gonzalez A, Hooper DU, Perrings C, Venail P, Narwani A, Mace GM, Tilman D, Wardle DA, Kinzig AP, Daily GC, Loreau M, Grace JB, Larigauderie A, Srivastava DS, Naeem S (2012). Biodiversity loss and its impact on humanity. Nature.

[45] Knapp S, Winter M, Klotz S (2017). Increasing species richness but decreasing phylogenetic richness and divergence over a 320-year period of urbanization. J Appl Ecol.

[46] Bissett A, Brown MV, Siciliano SD, Thrall PH (2013). Microbial community responses to anthropogenically induced environmental change: towards a systems approach. Ecol Lett.

[47] Dickson RP, Huffnagle GB (2015). The lung microbiome: new principles for respiratory bacteriology in health and disease. PLOS Pathog.

[48] Proctor DM, Relman DA (2017). The landscape ecology and microbiota of the human nose, mouth, and throat. Cell Host Microbe.

[49] Camarinha-Silva A, Jáuregui R, Pieper DH, Wos-Oxley ML (2012). The temporal dynamics of bacterial communities across human anterior nares. Environ Microbiol Rep.

[50] McMullen C, Alexander TW, Orsel K, Timsit E (2020). Progression of nasopharyngeal and tracheal bacterial microbiotas of feedlot cattle during development of Bovine Respiratory Disease. Vet Microbiol.

[51] Mcdaneld TG, Kuehn LA, Keele JW (2018). Evaluating the microbiome of two sampling locations in the nasal cavity of cattle with bovine respiratory disease complex (BRDC) 1. J Anim Sci.

[52] Dassanayake RP, Call DR, Sawant AA, Carol Casavant N, Weiser GC, Knowles DP (2010). *Bibersteinia trehalosi* inhibits the growth of *Mannheimia haemolytic*a by a proximity-dependent mechanism. Appl Environ Microbiol.

[53] Cortese VS, Braun DA, Crouch D, Townsend C, Zukowski B (2012). Case report—peracute to acute fatal pneumonia in cattle caused by *Bibersteinia trehalosi*. Bov Pract.

[54] Janda JM, Abbott SL (2007). 16S rRNA gene sequencing for bacterial identification in the diagnostic laboratory: pluses, perils, and pitfalls. J Clin Microbiol.

[55] Dabo SM, Taylor JD, Confer AW (2008). *Pasteurella multocida* and Bovine Respiratory Disease. Anim Heal Res Rev.

[56] Timsit E, Hallewell J, Booker C, Tison N, Amat S, Alexander TW (2017). Prevalence and antimicrobial susceptibility of *Mannheimia haemolytica, Pasteurella multocida*, and *Histophilus somni* isolated from the lower respiratory tract of healthy feedlot cattle and those diagnosed with Bovine Respiratory Disease. Vet Microbiol.

[57] Fulton RW, Blook KS, Panciera RJ, Confer AW, Ridpath JF, Saliki JT. Respiratory disease in feedlot cattle: isolation of infectious agents and lung lesions in fatal feedlot cases. Am Assoc Vet Lab Diagn. 2003;147. https://www.ars.usda.gov/research/publications/publication/?seqNo115=154572

[58] Agnes JT, Zekarias B, Shao M, Anderson ML, Gershwin LJ, Corbeil LB (2013). Bovine respiratory syncytial virus and *Histophilus somni* interaction at the alveolar barrier. Infect Immun.

[59] Caswell JL, Archambault M (1996). *Mycoplasma bovis* pneumonia in cattle. Anim Health Res Rev.

[60] Rice JA, Carrasco-Medina L, Hodgins DC, Shewen PE (2008). *Mannheimia haemolytica* and bovine respiratory disease. Anim Health Res Rev.

[61] Duff GC, Galyean ML (2007). Board-invited review: recent advances in management of highly stressed, newly received feedlot cattle. J Anim Sci.

[62] Timsit E, Bareille N, Seegers H, Lehebel A, Assié S (2011). Visually undetected fever episodes in newly received beef bulls at a fattening operation: occurrence, duration, and impact on performance. J Anim Sci.

[63] Waters CM, Bassler BL (2005). Quorum sensing: cell-to-cell communication in bacteria. Annu Rev Cell Dev Biol.

[64] Gentry TJ, Pepper IL, Pierson LS, Pepper IL, Gerba CP, Gentry TJ (2015). Microbial diversity and interactions in natural ecosystems. Environmental microbiology.

[65] Zeineldin M, Elolimy A, Barakat R (2020). Meta-analysis of bovine respiratory microbiota: link between respiratory microbiota and bovine respiratory health. FEMS Microbiol Ecol.

[66] Faner R, Sibila O, Agustí A, Bernasconi E, Chalmers JD, Huffnagle GB, Manichanh C, Molyneaux PL, Paredes R, Pérez Brocal V, Ponomarenko J, Sethi S, Dorca J, Monsó E (2017). The microbiome in respiratory medicine: current challenges and future perspectives. Eur Respir J.

[67] Amat S, Holman DB, Timsit E, Schwinghamer T, Alexander TW (2019). Evaluation of the nasopharyngeal microbiota in beef cattle transported to a feedlot, with a focus on lactic acid-producing bacteria. Front Microbiol.

[68] Holman DB, Timsit E, Alexander TW (2015). The nasopharyngeal microbiota of feedlot cattle. Sci Rep.

[69] Rosengarten R, Citti C, Glew M, Lischewski A, Droesse M, Much P, Winner F, Brank M, Spergser J (2000). Host-pathogen interactions in *Mycoplasma* pathogenesis: virulence and survival strategies of minimalist prokaryotes. Int J Med Microbiol.

[70] Kozich JJ, Westcott SL, Baxter NT, Highlander SK, Schloss PD (2013). Development of a dual-index sequencing strategy and curation pipeline for analyzing amplicon sequence data on the miseq illumina sequencing platform. Appl Environ Microbiol.

[71] Callahan BJ, McMurdie PJ, Rosen MJ, Han AW, Johnson AJA, Holmes SP (2016). DADA2: high-resolution sample inference from Illumina amplicon data. Nat Methods.

[72] Chao A (1984). Nonparametric estimation of the number of classes in a population. Scand J Stat.

[73] DeSantis TZ, Hugenholtz P, Larsen N, Rojas M, Brodie EL, Keller K (2006). Greengenes, a chimera-checked 16S rRNA gene database and workbench compatible with ARB. Appl Environ Microbiol.

[74] Pielou EC (1966). The measurement of diversity in different types of biological collections. J Theor Biol.

[75] Faith DP (1992). Conservation evaluation and phylogenetic diversity. Biol Conserv.

[76] Lozupone C, Knight R (2005). UniFrac: a new phylogenetic method for comparing microbial communities. Appl Environ Microbiol.

[77] Anders S, Huber W (2010). Differential expression analysis for sequence count data. Genome Biol.

[78] Camacho C, Coulouris G, Avagyan V, Ma N, Papadopoulos J, Bealer K (2009). BLAST+: architecture and applications. BMC Bioinformatics.

[79] Sachse K, Salam HSH, Diller R, Schubert E, Hoffmann B, Hotzel H (2010). Use of a novel real-time PCR technique to monitor and quantitate *Mycoplasma bovis* infection in cattle herds with mastitis and respiratory disease. Vet J.

[80] Guenther S, Schierack P, Grobbel M, Lübke-Becker A, Wieler LH, Ewers C (2008). Real-time PCR assay for the detection of species of the genus *Mannheimia*. J Microbiol Methods.

[81] Horwood PF, Mahony TJ (2007). Rapid detection of Bovine Respiratory Disease pathogens.

[82] Galkiewicz JP, Kellogg CA (2008). Cross-kingdom amplification using Bacteria-specific primers: complications for studies of coral microbial ecology. Appl Environ Microbiol.

[83] Leigh MB, Pellizari VH, Uhlík O, Sutka R, Rodrigues J, Ostrom NE (2007). Biphenyl-utilizing bacteria and their functional genes in a pine root zone contaminated with polychlorinated biphenyls (PCBs). ISME J.

[84] Singmann H, Bolker B, Westfall J, Aust F, Ben-Shachar MS, Højsgaardm S. Package “afex” analysis of factorial experiments. 2021.

[85] Oksanen J, Blanchet FG, Friendly M, Kindt R, Legendre P, Mcglinn D. Package “vegan” title community ecology package version 2.5-6. 2019.

[86] Griffith DM, Veech JA, Marsh CJ. Package “cooccur”, probabilistic species co-occurrence analysis in R. 2016.

