# Supplementary material for: Identification of bovine respiratory disease through the nasal microbiome
Source: Anim Microbiome. 2022 Feb 22;4:15. doi: 10.1186/s42523-022-00167-y (PMC8862248; doi:10.1186/s42523-022-00167-y)
Supplement: Supplementary file 1 — Additional File 1. Supplementary Results. Figure S1. Top 10 most abundant genera present in the DNA extraction negative controls used as a negative control in the DNA extraction and sequencing step. Figure S2. Holstein steer nasal microbiome alpha diversity relative to the date of collection. Figure S3. Variation in the cattle’s nasal alpha diversity estimated relative to the average daily temperature (°F). Figure S4. Average relative abundance of the 10 most abundant phyla (a), family (b), and genera (c) present in the nasal microbiome of BRD and healthy animals. Figure S5. Variable importance measured by random forest indicated by the mean decrease accuracy. Figure S6. Visual representation of the positive ASV co-occurrence analysis in the healthy group (n = 74) with a probability of occurrence in the same sample > 0.9. Figure S7. Visual representation of the positive ASV co-occurrence analysis in the BRD group (n = 57) with a probability of occurrence in the same sample > 0.9. Figure S8. Visual representation of the negative ASV co-occurrence analysis of the healthy group (n = 74) with a probability of occurrence in the same sample < 0.05. Figure S9. Visual representation of the negative ASV co-occurrence analysis in the BRD group (n = 57) with a probability of occurrence in the same sample < 0.05. Table S1. Variation in the cattle’s nasal alpha diversity relative to the animal age (months) analyzed using General Linear Mixed Model. Table S2. Sample average distance to the centroids of the BRD and healthy groups. Table S3. Prevalence of ASVs assigned as P. multocida, H. somni and M. bovis the nasal cavity of BRD (n = 75) and healthy (n = 74) groups based on 16S rRNA gene sequencing. Table S4. Prevalence of the genera Pasteurella, Histophilus, Mannheimia or Mycoplasma in the nasal cavity of Holstein steers (n=131) and between BRD (n = 75) and healthy (n = 74) animals based on 16S rRNA gene sequencing. Table S5. Positive ASV pair combinations present in 73 out of 7 [file 42523_2022_167_MOESM1_ESM.docx]

**ADDITIONAL FILE 1**

Identifying Bovine Respiratory Disease through the Nasal Microbiome

Ruth Eunice Centeno-Martinez, Natalie Glidden, Suraj Mohan, Josiah Levi Davidson, Esteban Fernández-Juricic, Jacquelyn P. Boerman, Jon Schoonmaker, Deepti Pillai, Jennifer Koziol, Aaron Ault, Mohit S. Verma, Timothy A. Johnson

**RESULTS**

**Mock community, DNA extraction negative controls analysis, and identification of contaminants.**

Three mock community samples were amplified and sequenced. From 648,634 sequences, a total of 54 amplicon sequence variants (ASVs) were identified in the three mock communities used in the study. After comparing the unknown ASVs to the 20 known bacteria strains, 18 out of 20 of the reference bacteria were identified. The contaminant ASVs in the mock community samples were rare and assigned to the class Chloroplast (abundance < 0.0001%), order *Ruminococcaceae* (0.001%), and species *Lysobacter enzymogenes* (< 0.0001%) and unclassified *Faecalibacterium* (0.0004%). For the DNA extraction negative controls, a total of 719 ASVs and 2,706,853 sequences were obtained in the study. 299 ASVs were shared between the DNA extraction negative controls and the swab samples. These shared ASVs were mostly composed at the family level by *Moraxellaceae* (5.016%), *Xanthomonadaceae* (4.34%), and *Burkolderiaceae* (3.67%). At the genus level by *Corynebacterium* 1 (2.67%), and *Strenotrophomonas* (2.67%); however, only two ASVs assigned to as either *Pseudoalteromonas* or *Vibrio* composed >10% of the community in DNA extraction negative controls (see Additional File 1: Figure S1). Because of this, these two ASVs were considered contaminants and were removed from all nasal swab samples. When comparing the ASVs obtained from the mock community and DNA extraction negative controls, no shared ASVs were observed.

**Nasal microbiome alpha diversity**

We found that time of year and animal age both impacted the alpha diversity of the nasal microbial community (see Additional File 1: Figure S2 and Table S1). Observed ASVs (F 1, 120.00= 18.740, *p* < 0.0001; Fig. 2a), Chao 1 (F 1, 120.00=16.5301, *p* < 0.0001; Fig. 2b), evenness (F 1, 119.99= 37.0722, *p* < 0.0001; Fig. 2c) and phylogenetic diversity (F 1,119.89=15.047, *p* < 0.0001; Fig. 2d), were all significantly lower in nasal samples collected later in the study. In regards to animal age, observed ASVs (F 1,119.87= 5.424, *p* <0.02), Chao 1 (F 1,119.70= 4.9954, *p* <0.02), evenness (F 1, 119.99=6.6816, *p* <0.01), and phylogenetic diversity (F 1,119.34=4.007, *p* <0.04) were all significantly positively associated with animal age (see Additional File 1: Figure S2).

One possible factor that could have contributed to the decrease in alpha diversity from the beginning to the end of the study is the environmental temperature. Samples were collected from July (summer) to December (winter) 2020. To identify if the environmental temperature could affect the difference in alpha diversity of the nasal cavity, the average temperature of the dates when the samples were collected was retrieved from wunderground.com. Average daily temperature data was retrieved from the closest weather station to the farm where the samples were collected. Correlation using Pearson’s correlation indicated a significant positive correlation between the average temperature and date when the samples were collected in this study (t 122=12.901; R=0.76, *p* < 0.0001). A general linear mixed model was performed using average daily temperature as a continuous factor and pen as random factor with random slope. The bacterial richness estimated by Observed ASVs (F 1, 110.82=7.4379, *p* <0.007) and Chao1 (F 1, 111.12=6.4313, *p* < 0.01), evenness estimated by Pielou_e (F 1, 112.5=13.595, *p* < 0.0004) and phylogenetic diversity estimated by Faith_pd (F 1, 115.68=5.4671, *p* < 0.021) The nasal alpha diversity metrics were positively associated with average temperature (see Additional File 1: Figure S3).


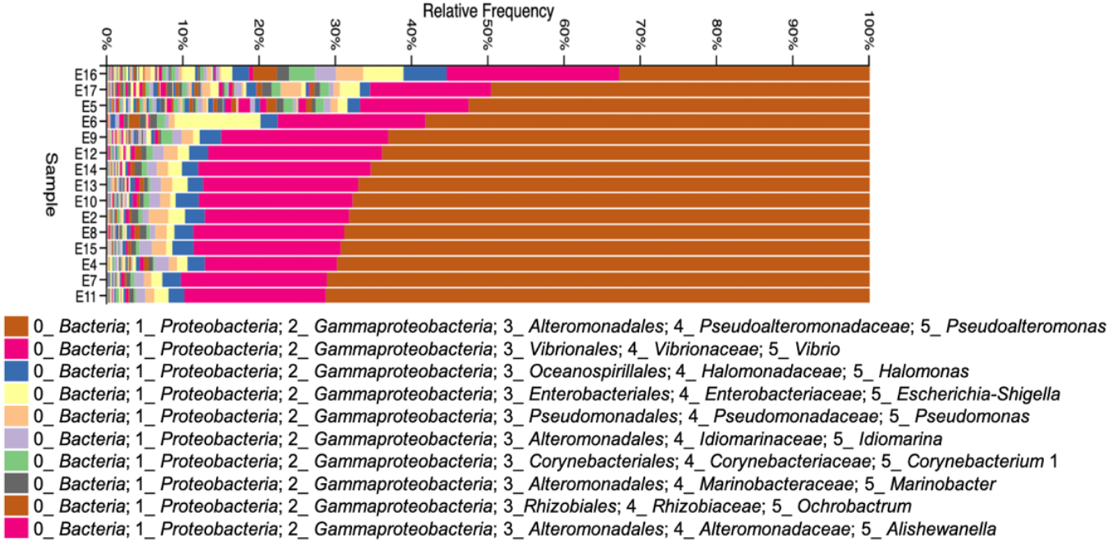


Figure S1. Top 10 most abundant genera present in the DNA extraction negative controls used as a negative control in the DNA extraction and sequencing step.


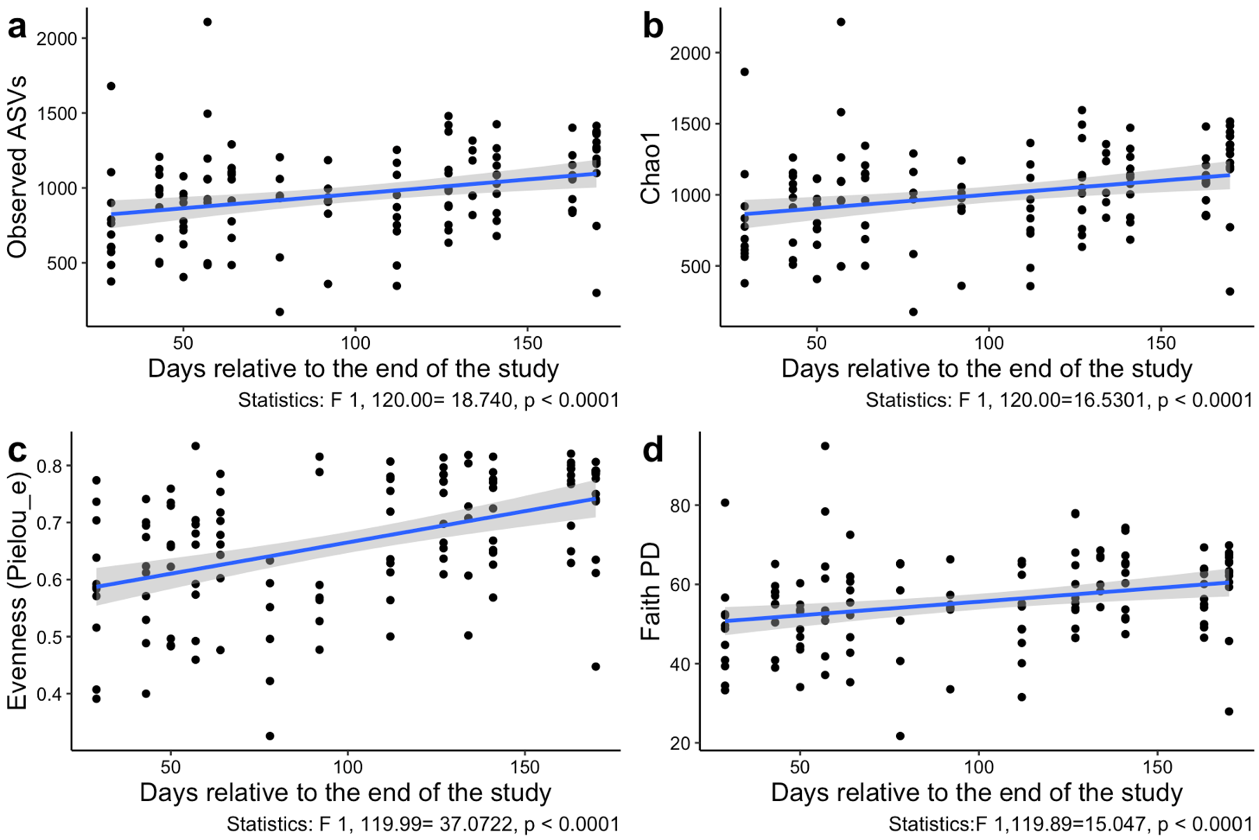


**Figure S2.** Holstein steer nasal microbiome alpha diversity relative to the date of collection. Variation in the cattle nasal microbiome alpha diversity estimated by Observed ASVs (**a**), Chao1 (**b**), Pielou (**c**) and Faith’s PD (**d**) (± 95% confident bands) relative to the date of collection. High values in the x axis represent the dates of samples collected at the beginning of the study (July, 2020); low values represent the dates of samples collected at the end of the study (December, 2020). Alpha diversity metrics measured using General Linear Mixed Model.


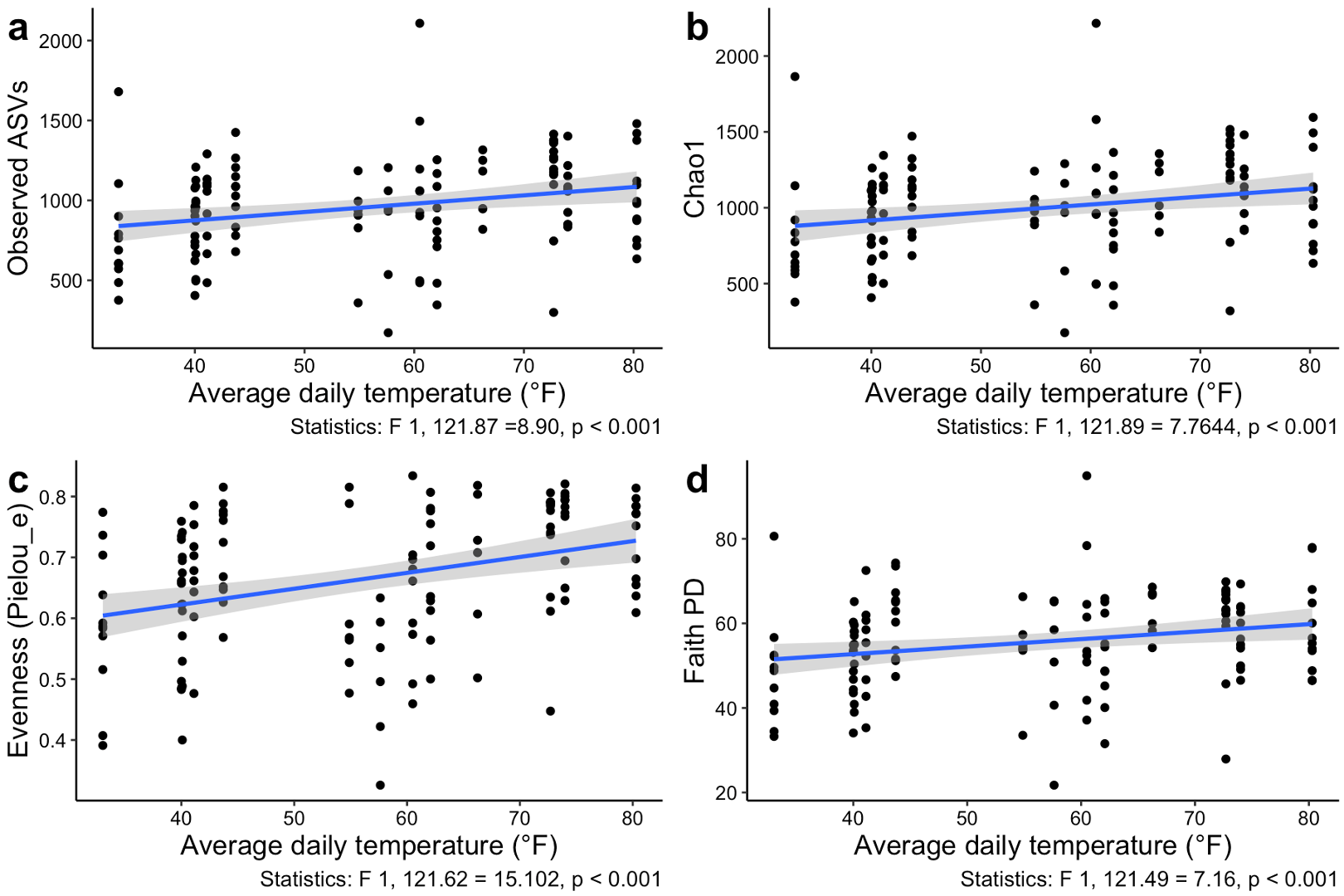


**Figure S3.** Variation in the cattle’s nasal alpha diversity estimated relative to the average daily temperature (°F). Variation in the cattle nasal alpha diversity estimated by Observed ASVs (**a**), Chao1 (**b**), Pielou (**c**) and Faith’s PD (**d**) (± 95% confident bands). Alpha diversity metrics measured using General Linear Mixed Model.

**
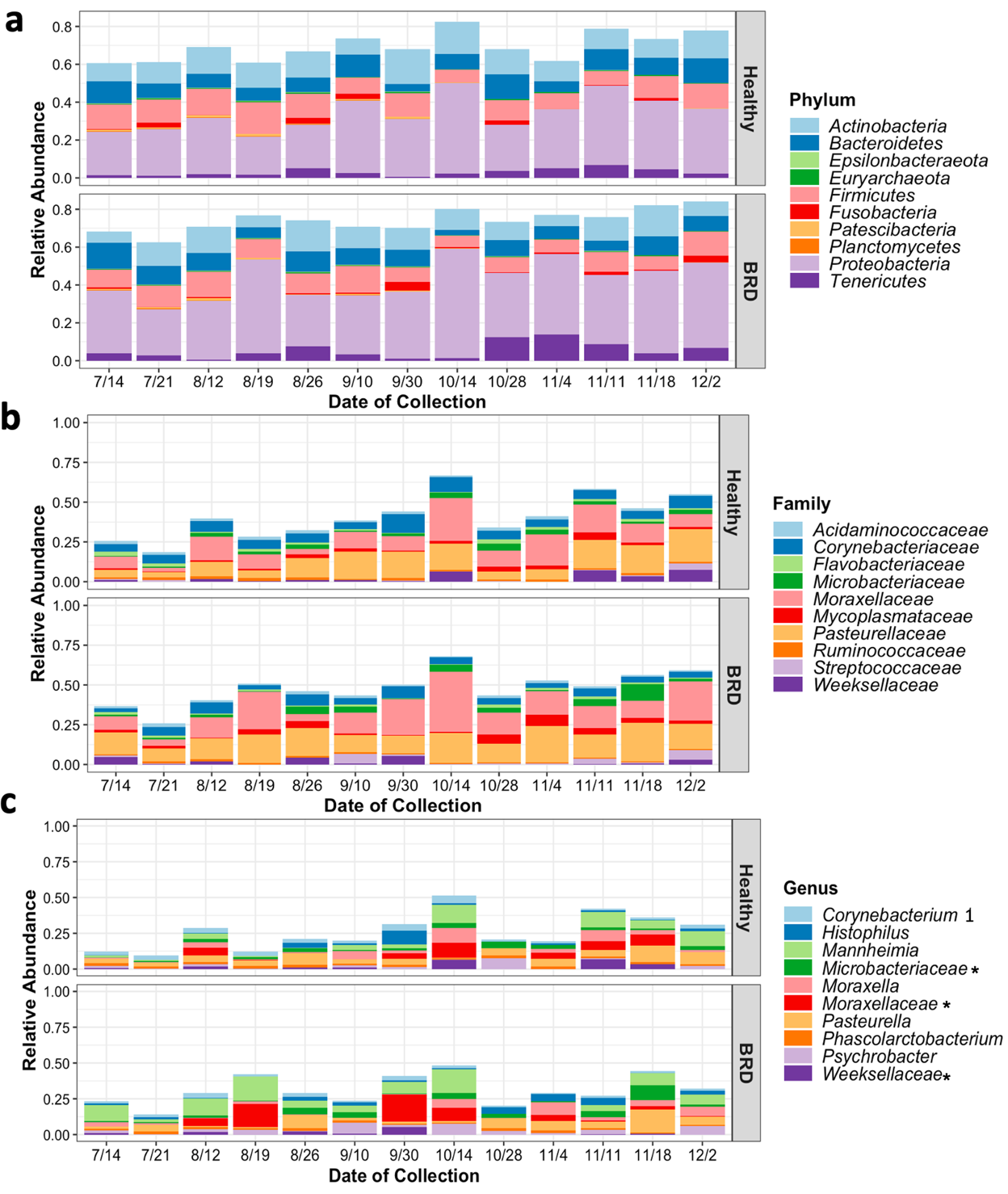
**

**Figure S4.** Average relative abundance of the 10 most abundant phyla (**a**), family (**b**) and genera (**c**) present in the nasal microbiome of BRD and healthy animals. * represents ASVs there were unclassified at the genus level.

**
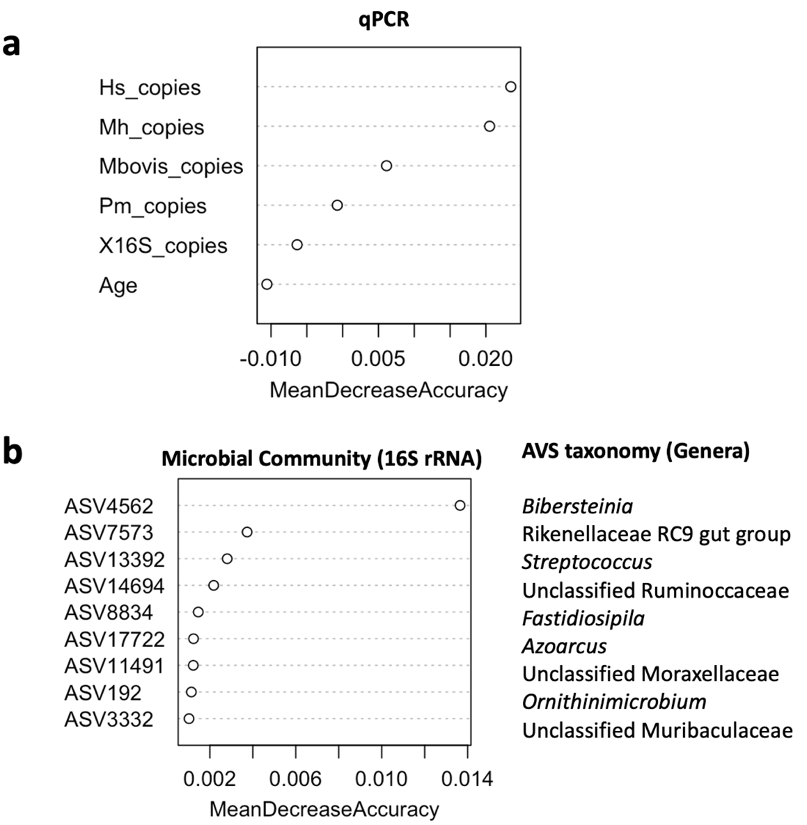
**

**Figure S5.** Variable importance measured by random forest indicated by the mean decrease accuracy. Variable importance measured the abundance of BRD pathobionts, 16S rRNA gene abundance and animal age (qPCR) (**a**) or by using microbial community composition determined by 16S rRNA gene sequencing (**b**).


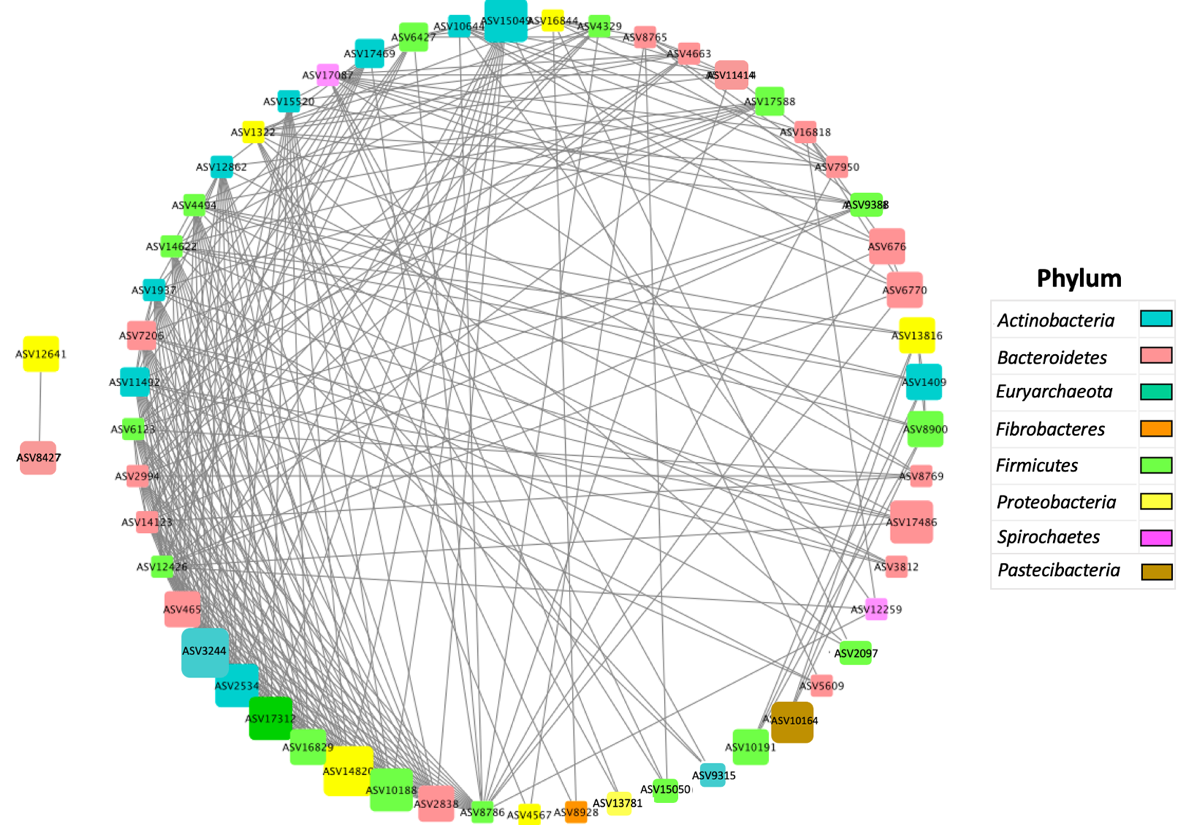


Figure S6. Visual representation of the positive ASV co-occurrence analysis in the healthy group (n=74) with a probability of occurrence in the same sample > 0.9. Box sizes represent the total samples where the co-occurrence was observed (min value 69 and max value 73 samples). Color indicates the phylum classification. Lines indicate co-occurrence between ASVs.


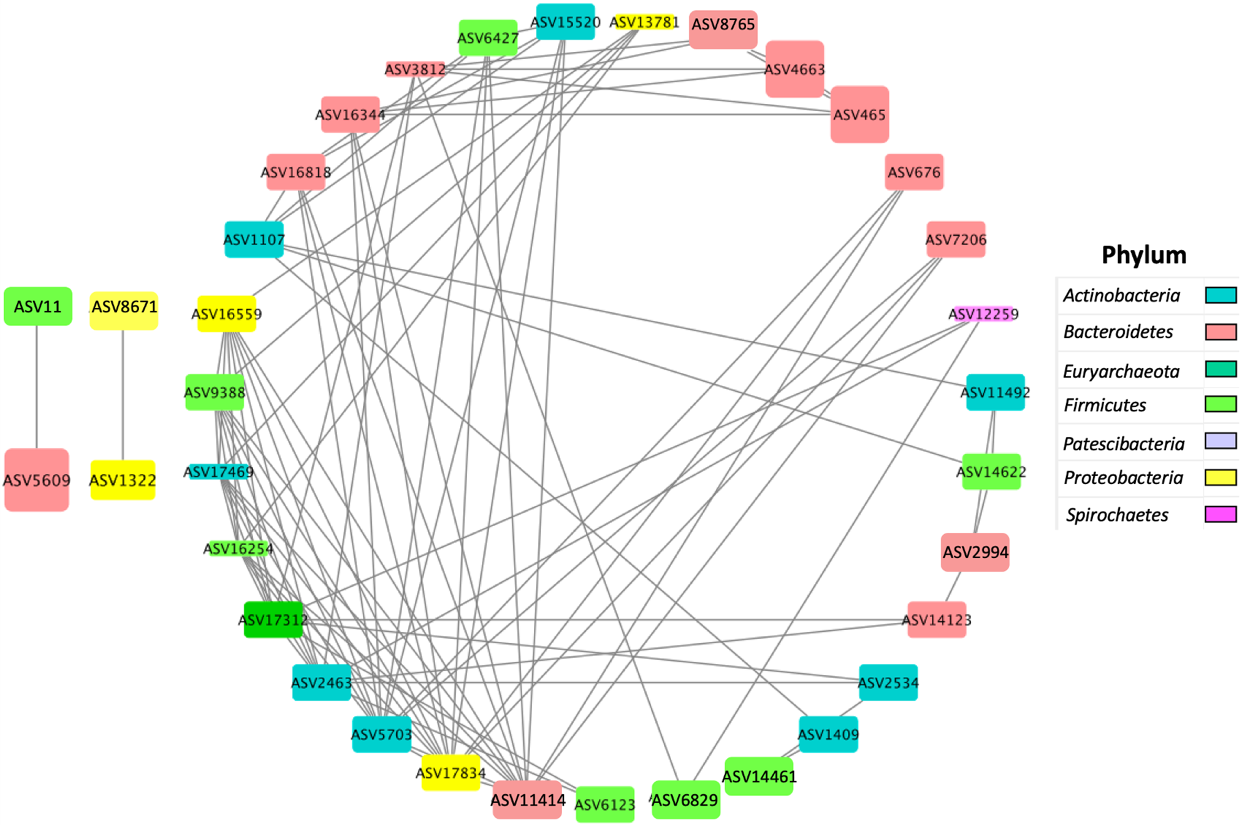


Figure S7. Visual representation of the positive ASV co-occurrence analysis in the BRD group (n=57) with a probability of occurrence in the same sample > 0.9. Box sizes represent the total samples where the co-occurrence was observed (min value 54 and max value 56 samples). Color indicates the phylum classification. Lines indicate co-occurrence between ASVs.


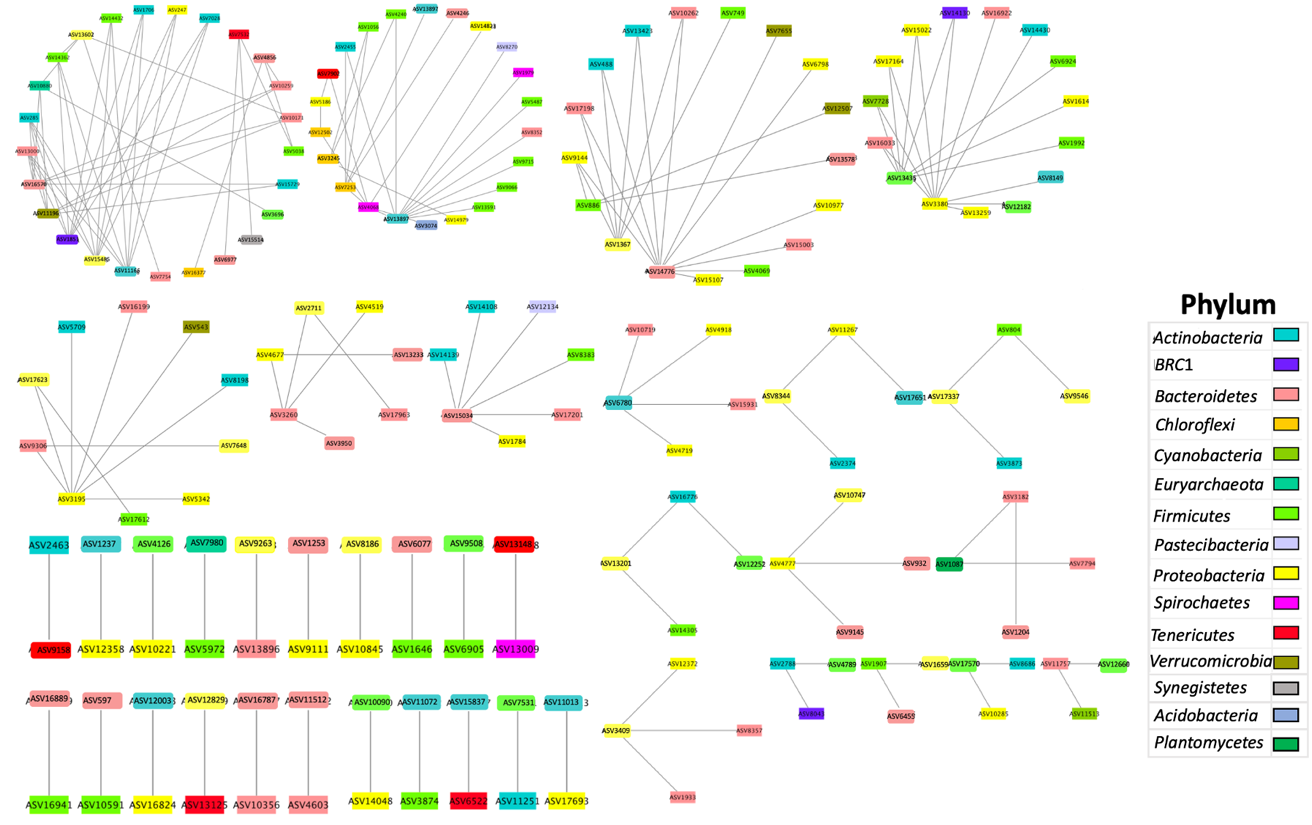


Figure S8. Visual representation of the negative ASV co-occurrence analysis of the healthy group (n=74) with a probability of occurrence in the same sample < 0.05. Color indicates the phylum classification. Lines indicate negative associations between ASVs.


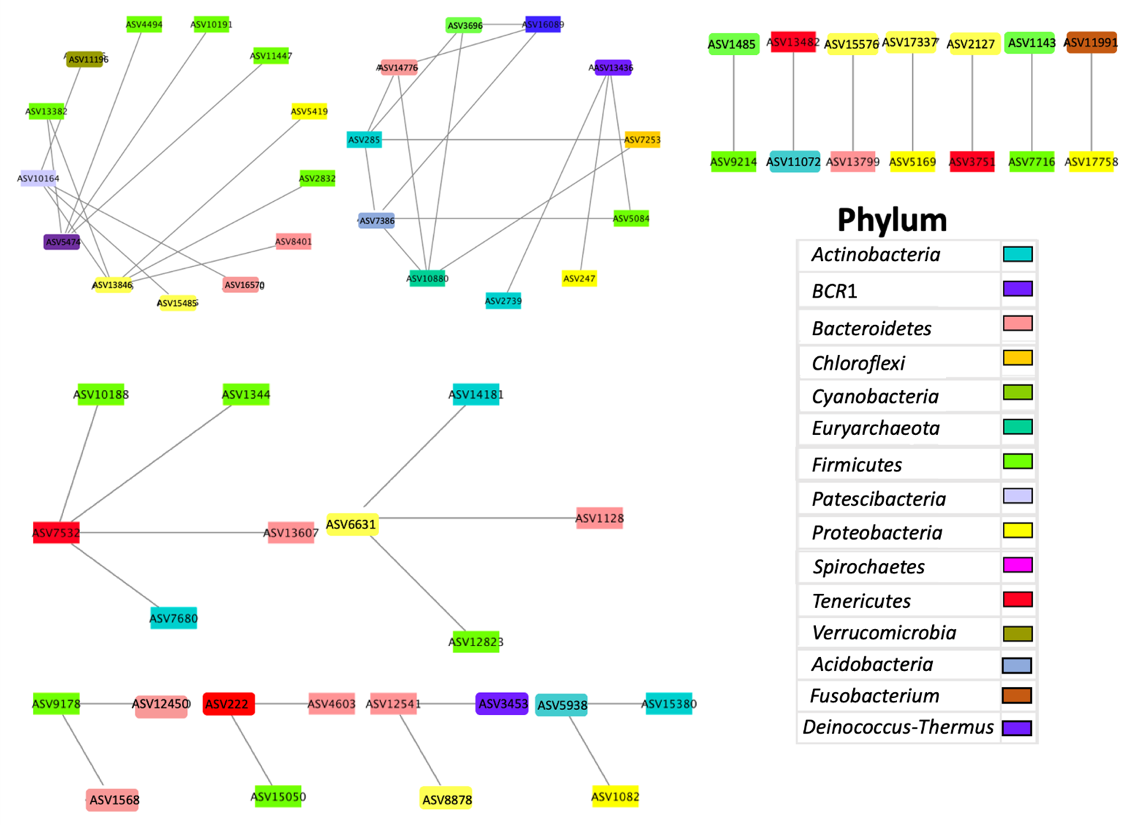


Figure S9. Visual representation of the negative ASV co-occurrence analysis in the BRD group (n=57) with a probability of occurrence in the same sample < 0.05. Color indicates the phylum classification. Lines indicate negative associations between ASVs.

**Table S1.** Variation in the cattle’s nasal alpha diversity relative to the animal age (months) analyzed using General Linear Mixed Model.

|  |  | **Age** |  |
| --- | --- | --- | --- |
| **Alpha diversity metric** | **DF** | ***F-*Value** | ***P-*Value** |
| Observed ASVs | 1, 119.87 | 5.42 | <0.02 |
| Chao 1 | 1, 119.70 | 4.99 | <0.02 |
| Pielou_e | 1, 119.99 | 6.68 | <0.01 |
| Faith_PD | 1, 119.34 | 4.00 | <0.04 |

**Table S2**. Sample average distance to the centroids of the BRD and healthy groups.

| **Beta diversity metric** | **BRD** | **Healthy** | ***P-*Value** |
| --- | --- | --- | --- |
| Distance (Bray-Curtis) | 0.5001 | 0.4743 | 0.058 |
| Distance (Weighted UniFrac) | 0.3369 | 0.3099 | 0.085 |

Table S3. Prevalence of ASVs assigned as *P. multocida, H. somni* and *M. bovis* the nasal cavity of BRD (n=75) and healthy (n=74) groups based on 16S rRNA gene sequencing.

|  | ***P. multocida*** | ***M. haemolytica*** | ***M. bovis* ASV13707** | ***M. bovis* ASV13464** | ***M. bovis* ASV2171** | ***M. bovis* ASV15117** | ***M. bovis* ASV10207** |
| --- | --- | --- | --- | --- | --- | --- | --- |
| BRD | 55 (96%) | 1 (2%) | 37 (65%) | 1 (2%) | 2 (4%) | 0 (0%) | 5 (9%) |
| Healthy | 72 (97%) | 0 (0%) | 30 (41%) | 0 (0%) | 1 (1%) | 1 (1%) | 1 (1%) |

**Table S4.** Prevalence of the genera *Pasteurella, Histophilus, Mannheimia* or *Mycoplasma* in the nasal cavity of Holstein steers (n=131) and between BRD (n=75) and healthy (n=74) animals based on 16S rRNA gene sequencing.

|  | ***Pasteurella*** | ***Histophilus*** | ***Mannheimia*** | ***Mycoplasma*** |
| --- | --- | --- | --- | --- |
| BRD | 55 (96.5%) | 45 (78.9%) | 37 (64.9%) | 53 (93%) |
| Healthy | 72 (97.3%) | 55 (74.3%) | 43 (58.1%) | 60 (81.1%) |
| All | 127 (96.94%) | 100 (76.33%) | 80 (61.06%) | 113 (86.25%) |

Table S5. Positive ASV pair combinations present in 73 out of 74 healthy samples with a probability of occurrence in the same sample > 0.9.

| **ASVs 1** | **Genus_1** | **ASVs 2** | **Genus_2** |
| --- | --- | --- | --- |
| ASV10188 | *Romboutsia* | ASV16829 | *Guggenheimella* |
| ASV10188 | *Romboutsia* | ASV2838 | *Prevotellaceae* NK3B31 group |
| ASV10188 | *Romboutsia* | ASV465 | *Prevotellaceae* NK3B31 group |
| ASV1409 | *Georgenia* | ASV13816 | *Parapusillimonas* |
| ASV1409 | *Georgenia* | ASV8900 | *Uncultured Acidaminococcaceae* |
| ASV14820 | *Acinetobacter* | ASV10188 | *Romboutsia* |
| ASV14820 | *Acinetobacter* | ASV16829 | *Guggenheimella* |
| ASV14820 | *Acinetobacter* | ASV17312 | *Methanobrevibacter* |
| ASV14820 | *Acinetobacter* | ASV2534 | *Olsenella* |
| ASV14820 | *Acinetobacter* | ASV2838 | *Prevotellaceae* NK3B31 group |
| ASV14820 | *Acinetobacter* | ASV465 | *Prevotellaceae* NK3B31 group |
| ASV16829 | *Guggenheimella* | ASV2838 | *Prevotellaceae* NK3B31 group |
| ASV16829 | *Guggenheimella* | ASV465 | *Prevotellaceae* NK3B31 group |
| ASV17312 | *Methanobrevibacter* | ASV10188 | *Romboutsia* |
| ASV17312 | *Methanobrevibacter* | ASV16829 | *Guggenheimella* |
| ASV17312 | *Methanobrevibacter* | ASV2534 | *Olsenella* |
| ASV17312 | *Methanobrevibacter* | ASV2838 | *Prevotellaceae* NK3B31 group |
| ASV17312 | *Methanobrevibacter* | ASV465 | *Prevotellaceae* NK3B31 group |
| ASV2534 | *Olsenella* | ASV10188 | *Romboutsia* |
| ASV2534 | *Olsenella* | ASV16829 | *Guggenheimella* |
| ASV2534 | *Olsenella* | ASV2838 | *Prevotellaceae* NK3B31 group |
| ASV2534 | *Olsenella* | ASV465 | *Prevotellaceae* NK3B31 group |
| ASV3244 | *Corynebacterium* 1 | ASV10188 | *Romboutsia* |
| ASV3244 | *Corynebacterium* 1 | ASV14820 | *Acinetobacter* |
| ASV3244 | *Corynebacterium* 1 | ASV16829 | *Guggenheimella* |
| ASV3244 | *Corynebacterium* 1 | ASV17312 | *Methanobrevibacter* |
| ASV3244 | *Corynebacterium* 1 | ASV2534 | *Olsenella* |
| ASV3244 | *Corynebacterium* 1 | ASV2838 | *Prevotellaceae* NK3B31 group |
| ASV3244 | *Corynebacterium* 1 | ASV465 | *Prevotellaceae* NK3B31 group |
| ASV465 | *Prevotellaceae* NK3B31 group | ASV2838 | *Prevotellaceae* NK3B31 group |
| ASV676 | *Prevotellaceae* NK3B31 group | ASV6770 | *Fermentimonas* |
| ASV8900 | Uncultured *Acidaminococcaceae* | ASV13816 | *Parapusillimonas* |

Table S6. Co-occurrence analysis with positive ASV pair combinations present in 56 out of 57 BRD samples with a probability of occurrence in the same sample of 1.

| **ASV 1** | **Genus_1** | **ASV 2** | **Genus_2** |
| --- | --- | --- | --- |
| ASV11414 | *Flavobacterium* | ASV17834 | *Halomonas* |
| ASV5703 | *Glutamicibacter* | ASV17834 | *Halomonas* |
| ASV17312 | *Methanobrevibacter* | ASV2463 | *Gulosibacter* |
| ASV8765 | *Prevotella* 1 | ASV465 | *Prevotellaceae* NK3B31 group |
| ASV465 | *Prevotellaceae* NK3B31 group | ASV4663 | *Prevotellaceae* NK3B31 group |
| ASV8765 | *Prevotella* 1 | ASV4663 | *Prevotellaceae* NK3B31 group |
| ASV11 | *Clostridium sensu stricto* 1 | ASV5609 | *Bacteroides* |
| ASV11414 | *Flavobacterium* | ASV5703 | *Glutamicibacter* |

Table S7. Co-occurrence analysis showing negative ASV pair combinations that are not likely to co-exist in the heathy group with a probability <0.04.

| **ASV 1** | **Genus_1** | **ASV 1** | **Genus_2** |
| --- | --- | --- | --- |
| ASV4603 | uncultured *Bacteroides* sp*.* | ASV11512 | uncultured *Prevotellaceae bacterium* |
| ASV2463 | uncultured *Gulosibacter* | ASV9158 | *Mycoplasma bovirhinis* |
| ASV285 | uncultured *Bifidobacteriaceae* | ASV11166 | *Intrasporangiaceae* |
| ASV13000 | *Prevotellaceae* UCG-003 | ASV11166 | *Intrasporangiaceae* |
| ASV14432 | uncultured *Guggenheimella* | ASV11166 | *Intrasporangiaceae* |
| ASV14432 | uncultured *Guggenheimella* | ASV15485 | *Thermomonas* |
| ASV13000 | *Prevotellaceae* UCG-003 | ASV15485 | *Thermomonas* |
| ASV285 | uncultured *Bifidobacteriaceae* | ASV15485 | *Thermomonas* |
| ASV13000 | *Prevotellaceae* UCG-003 | ASV1851 | uncultured *Desulfocaldus* sp*.* |
| ASV285 | uncultured *Bifidobacteriaceae* | ASV1851 | uncultured *Desulfocaldus* sp. |
| ASV14432 | uncultured *Guggenheimella* | ASV1851 | uncultured *Desulfocaldus* sp*.* |
| ASV1706 | *Corynebacterium* 1 | ASV11166 | *Intrasporangiaceae* |
| ASV14362 | uncultured *Lysinibacillus* | ASV11166 | *Intrasporangiaceae* |
| ASV1706 | *Corynebacterium* 1 | ASV15485 | *Thermomonas* |
| ASV14362 | uncultured *Lysinibacillus* | ASV15485 | *Thermomonas* |
| ASV14362 | uncultured *Lysinibacillus* | ASV1851 | uncultured *Desulfocaldus* sp*.* |
| ASV1706 | *Corynebacterium* 1 | ASV1851 | uncultured *Desulfocaldus* sp*.* |
| ASV7028 | *Arthrobacter* | ASV11166 | *Intrasporangiaceae* |
| ASV247 | uncultured *Oligella* | ASV11166 | *Intrasporangiaceae* |
| ASV12358 | uncultured *Cellvibrio* | ASV1237 | *Microbacteriaceae* |
| ASV7028 | *Arthrobacter* | ASV15485 | *Thermomonas* |
| ASV247 | uncultured *Oligella* | ASV15485 | *Thermomonas* |
| ASV247 | uncultured *Oligella* | ASV1851 | uncultured *Desulfocaldus* sp. |
| ASV7028 | *Arthrobacter* | ASV1851 | uncultured *Desulfocaldus* sp. |
| ASV10880 | *Methanobrevibacter* | ASV11196 | uncultured *Chthoniobacteraceae* |
| ASV10880 | *Methanobrevibacter* | ASV13602 | *Paracoccus* |
| ASV7532 | *Mycoplasma bovirhinis* | ASV15514 | *uncultured Synergistaceae* |
| ASV10880 | *Methanobrevibacter* | ASV16570 | *Flavobacterium* |
| ASV5038 | *Aerococcus* | ASV4856 | *Prevotellaceae* |
| ASV7532 | *Mycoplasma bovirhinis* | ASV5038 | *Aerococcus* |
| ASV7532 | *Mycoplasma bovirhinis* | ASV6977 | *Taibaiella* |
| ASV804 | *Ruminococcus* 1 | ASV9546 | *Vitreoscilla* |
| ASV10259 | *Chishuiella* sp. YIM 102668 | ASV11196 | uncultured *Chthoniobacter* |
| ASV10259 | *Chishuiella* sp. YIM 102668 | ASV16570 | *Flavobacterium* |
| ASV10259 | *Chishuiella* sp. YIM 102668 | ASV4856 | *Prevotellaceae* |
| ASV7754 | *Paludibacter* | ASV13602 | *Paracoccus* |

Table S8. Co-occurrence analysis showing negative ASV pair combinations that are not likely to co-exist in the BRD group with a probability <0.04.

| **ASV_1** | **Genus_1** | **ASV_1** | **Genus_2** |
| --- | --- | --- | --- |
| ASV10164 | uncultured *Absconditabacteriales* (SR1) | ASV11196 | uncultured *Chthoniobacter* |
| ASV10164 | uncultured *Absconditabacteriales* (SR1) | ASV15485 | *Thermomonas* |
| ASV10164 | uncultured *Absconditabacteriales* (SR1) | ASV16570 | *Flavobacterium* |
| ASV15050 | uncultured *Jeotgalibaca* | ASV222 | *Mycoplasma* |
| ASV7680 | *Corynebacterium* sp. C3 | ASV7532 | *Mycoplasma bovirhinis* |
| ASV1344 | *Planococcaceae* | ASV7532 | *Mycoplasma bovirhinis* |
| ASV13607 | *Sphingobacterium jejuense* | ASV7532 | *Mycoplasma bovirhinis* |
| ASV7716 | *Weissella paramesenteroides* | ASV1143 | uncultured *Murdochiella* |
| ASV3751 | *Mycoplasma bovoculi* M165/69 | ASV2127 | *Moraxella boevrei* DSM 14165 |
| ASV4603 | uncultured *Bacteroides* sp. | ASV222 | *Mycoplasma* |
| ASV12541 | uncultured *Rikenellaceae* RC9 | ASV3453 | *Deinococcus* |
| ASV10188 | *Romboutsia* | ASV7532 | *Mycoplasma bovirhinis* |
| ASV12541 | uncultured *Rikenellaceae* RC9 | ASV8878 | *Moraxellaceae* |

Table S9. Evaluation of *Pasteurella multocida, Histophilus somni, Mannheimia haemolytica* and *Mycoplasma bovis* using qPCR assays.

| **qPCR assay** |  | ***Pasteurella multocida*** | ***Histophilus somni*** | ***Mannheimia haemolytica*** | ***Mycoplasma bovis*** | **16S rRNA gene** |
| --- | --- | --- | --- | --- | --- | --- |
| Standard equation | Slope | -3.3 | -3.596 | -3.145 | -3.343 | -3.011 |
|  | Intercept | 36.921 | 39.723 | 35.92 | 37.098 | 35.77 |
|  | Replicates | 3 | 3 | 3 | 3 | 3 |
| Efficiency (%) |  | 100.94 | 89.71 | 107.96 | 99.13 | 114.84 |
| Dilutions |  | 9 | 9 | 9 | 9 | 9 |
| Cq cut-off value^Ψ^ |  | 33.53 | 36.77 | 31.76 | 32.1 | 30.52 |
| ^Ψ^ Cq value corresponding to the last dilution in the standard curve (10^0^) at which samples tested positive. | | | | | | |
